# Protocol and programme factors associated with referral and loss to follow-up from newborn hearing screening: a systematic review

**DOI:** 10.1186/s12887-022-03218-0

**Published:** 2022-08-05

**Authors:** Allison R. Mackey, Andrea M. L. Bussé, Valeria Del Vecchio, Elina Mäki-Torkko, Inger M. Uhlén

**Affiliations:** 1 Karolinska Institutet, Department of Clinical Science Intervention and Technology, Division of Ear, Nose and Throat Diseases, Karolinska University Hospital, Huddinge, 141 86 Stockholm, Sweden; 2 Department of Otorhinolaryngology and Head and Neck Surgery and Department of Ophthalmology, Erasmus University Medical Center, Rotterdam, The Netherlands; 3 Department of Neuroscience, University of Padua, Bologna, Italy; 4 Unit of Audiology, Department of Neuroscience, Reproductive Sciences and Dentistry, University of Naples Federico II, Naples, Italy; 5 Audiological Research Centre, Faculty of Medicine and Health, Örebro University, Örebro, Sweden; 6 School of Medical Sciences, Faculty of Medicine and Health, Örebro University, Örebro, Sweden

**Keywords:** Newborn hearing screening, Childhood hearing impairment, Early detection and intervention, Referral, Lost to follow-up, Quality assessment, Automated auditory brainstem response, Otoacoustic emissions

## Abstract

**Background:**

An effective newborn hearing screening programme has low referral rate and low loss to follow-up (LTFU) rate after referral from initial screening. This systematic review identified studies evaluating the effect of protocol and programme factors on these two outcomes, including the screening method used and the infant group.

**Methods:**

Five databases were searched (latest: April 2021). Included studies reported original data from newborn hearing screening and described the target outcomes against a protocol or programme level factor. Studies were excluded if results were only available for one risk condition, for each ear, or for < 100 infants, or if methodological bias was observed. Included studies were evaluated for quality across three domains: sample, screening and outcome, using modified criteria from the Ottawa-Newcastle and QUADAS-2 scales. Findings from the included studies were synthesised in tables, figures and text.

**Results:**

Fifty-eight studies reported on referral rate, 8 on LTFU rate, and 35 on both. Only 15 studies defined LTFU. Substantial diversity in referral and LTFU rate was observed across studies. Twelve of fourteen studies that evaluated screening method showed lower referral rates with aABR compared to TEOAE for well babies (WB). Rescreening before hospital discharge and screening after 3 days of age reduced referral rates. Studies investigating LTFU reported lower rates for programmes that had audiologist involvement, did not require fees for step 2, were embedded in a larger regional or national programme, and scheduled follow-up in a location accessible to the families. In programmes with low overall LTFU, higher LTFU was observed for infants from the NICU compared to WB.

**Conclusion:**

Although poor reporting and exclusion of non-English articles may limit the generalisability from this review, key influential factors for referral and LTFU rates were identified. Including aABR in WB screening can effectively reduce referral rates, but it is not the only solution. The reported referral and LTFU rates vary largely across studies, implying the contribution of several parameters identified in this review and the context in which the programme is performed. Extra attention should be paid to infants with higher risk for hearing impairment to ensure their return to follow-up.

**Supplementary Information:**

The online version contains supplementary material available at 10.1186/s12887-022-03218-0.

## Introduction

To detect permanent hearing impairment (PHI) and provide early intervention, newborn hearing screening (NHS) has become part of standard neonatal care in many countries around the world. Early detection and intervention leads to longstanding benefits in speech and language development [1]. A successful and cost-effective NHS programme detects all infants with PHI as early as possible (high sensitivity), and infants without PHI should pass screening (high specificity). A programme with high specificity reduces unnecessary stress on parents, burden on diagnostic clinics and higher costs associated with more diagnostic assessments.

Two important elements in a cost-effective NHS programme are low referral rates from screening and low loss to follow-up (LTFU) after referral [2]. The prevalence of PHI (0.1 to 0.2%) [3, 4] is magnitudes lower than referral rate from step 1 (2 to 22%) [5], so a low referral rate from screening generally indicates good specificity. Low LTFU is required to achieve good sensitivity of a screening programme, as infants with potential PHI are not lost after referral from screening [2]. In this systematic review, we will identify and evaluate the key protocol and programme factors that influence two NHS performance outcomes: the referral rate from screening step 1 and the LTFU rate after referral from screening step 1.

**Fig. 1:**
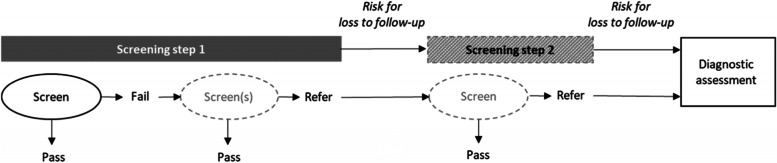
An example newborn hearing screening pathway. Infants are screened one or multiple times during screening step 1. Depending on the protocol, infants who are referred from screening step 1 may undergo screening step 2 or be directly referred to a diagnostic assessment

NHS is performed using one or more screening steps. Figure 1 displays an example screening pathway with the terms used in this article. Step 1 may be performed as either an inpatient (i.e., before maternity ward discharge) or as an outpatient (i.e., after maternity ward discharge). In each step, one or multiple screens may occur. The timing between screens within step 1 may vary depending on the protocol. Families of infants who fail step 1 are asked to return to a follow-up appointment, which may either be screening step 2 or a diagnostic assessment depending on the protocol. During the period after referral from step 1, there is a risk that families do not return to their follow-up appointment.

At step 1, one or two test methods may be used, otoacoustic emissions (OAEs) or automatic auditory brainstem response (aABR). Historically, both OAE and aABR have high sensitivity and specificity when performed under ideal conditions. However, the conditions in the field under which these tests are performed may vary depending on the protocol or situation (e.g., age of the infant, noisy setting, or inexperienced screeners). aABR screening is recommended among infants admitted to the neonatal intensive care unit (NICU) due to the higher prevalence of auditory neuropathy in this population. For well babies (WB), most programmes currently use only OAE screening for step 1 [6]. Though, aABR is becoming an appealing option for WB, either as a single screening method or in sequence directly after a failed OAE screen. If aABR screening can reduce the number of infants referred from step 1, this may be beneficial for hospitals with shorter maternity ward stays and particularly programmes struggling with high LTFU [2].

High LTFU is especially concerning among infants with a high risk for PHI. This group includes infants admitted to the NICU plus infants with risk factors (RF) for PHI [7], such as a family history of PHI and certain syndromes. It is unclear if high-risk infants (i.e., NICU/RF) are more or less susceptible to LTFU compared to WB/no RF [8]. For example, other medical diagnoses may take priority over hearing, and caregivers may have to travel longer to return to the screening hospital. Conversely, caregivers of an infant with a higher risk for PHI may be more informed on the effects of PHI and be more inclined to attend follow-up appointments. By understanding the relationship between infant risk status for PHI and LTFU, professionals and policymakers can better align quality improvement plans to their local programmes.

NHS protocols and programme features vary across countries and regions. This diversity includes the screening method used (OAE, aABR, or both), the screening professional, the age of the infant at screening, and the location where screening takes place [6]. Variation in reported referral and LTFU rates were also found across NHS programmes [5]. During implementation and quality improvement plans, it is important for NHS policymakers or expert groups to consider the factors associated with the protocol or programme that will affect the number of infants referred and the number of infants LTFU after referral from screening.

The primary aim of this study is to examine the published literature to identify the factors of an NHS programme that have an impact on referral and LTFU rate. Specifically, we investigate the screening method, including use of aABR in step 1, to reduce referral rates from step 1, as well as other determinants that can be modified or accounted for when organizing and managing NHS. The secondary aim is to determine whether infants with a higher risk for PHI also have a higher risk of being LTFU after screening. The findings of this systematic review will help inform expert groups or policymakers involved in NHS implementation, by describing the important associations of various NHS parameters on two key performance outcomes. Findings will also help guide strategies for performing quality improvements to improve NHS outcomes.

## Methods

This review followed the PRISMA framework for systematic reviews and meta-analyses [9]. A protocol was published on PROSPERO (registration number CRD42020155348).

### Literature search and selection of studies

Two medical librarians searched five databases (Medline Ovid, Embase, Cochrane Library, Web of Science Core Collection and Cinahl). The initial search was performed on May 15th 2019, and the search was updated on April 9th 2021. Search terms included a combination of MESH terms and free text. The search strategy combined three main concepts: hearing or hearing disorders; screening or technologies used for screening; and infant, newborn, nurseries or NICU. The complete search strategy for Ovid Medline is available in Additional file 1, which was adapted appropriately for each database. Filters for language or publication date were not applied within the search. Duplicates were removed, and the remaining records were imported into Endnote X9 for review.

The titles and abstracts of all records found through the search were examined by three independent reviewers (AM, VDV, and AB). Included records had titles and/or abstracts that referred to population-based newborn hearing screening. Records were excluded if: the report was not written in English; it was not peer-reviewed; screening on newborns was not performed; screening was not for hearing impairment; or screening was only performed on children already diagnosed with a hearing disorder. Records were included even if programme determinants or the outcomes in question were not mentioned in the abstract, because it was possible that they were present only in the body of the text. Any discrepancies in title/abstract sorting were resolved by majority decision. Data from experts and supported by grey literature (local NHS reports, student theses, etc) were recently aggregated across 47 countries or regions and published separately [5], and therefore expert consultations or grey literature were not included in this review.

Candidate reports underwent full-text sorting by two independent reviewers (AM and either VDV or AB). Reports were included if they described one or more programme determinants, as well as the referral rate from initial screening and/or follow-up or LTFU after a screening referral. Reports were excluded if: original data were not reported, screening was performed with techniques other than OAE or ABR; the number of infants screened was not identified; the sample comprised only infants with one or more specific conditions (e.g., hyperbilirubinemia); the infants screened were older than 6 months of age (exception: NICU infants); or results were only presented on the number of ears. Reports were excluded if the sample size per group was less than 100. Recently published step 1 referral rates ranged from 2 to 22% [5], therefore a sample size less than 100 lacks validity. Reports were excluded if the methodology was a descriptive, non-comparative case study. For example, implementation studies offering results from a single protocol and programme design with no comparison groups were excluded. All other study designs (i.e., observational cohort, random or non-randomised control trials) were eligible for inclusion. Discrepancies between reviewers were discussed until a consensus was made.

### Quality evaluation

All reports included from the full-text review underwent quality evaluation. In cases where the same data were presented in two reports, such as a pilot study plus follow-up study, the later published report was used for evaluation and analysis; however, information was drawn from earlier published reports if needed for evaluation. In cases where reports were published as a series on the same data, the reports were considered collectively as a single study.

A quality evaluation checklist was derived using modified criteria from the Newcastle-Ottawa scale for cohort studies [10] and QUADAS-2 scale [11]. Because some of the criteria in the original scales were not applicable to the research questions, criteria were adapted. The modified scale is presented in Table 1. From these criteria, we identified four that were deemed essential to achieve internal validity. Studies that did not meet all four essential criteria were determined to have a risk of bias in the outcome and were excluded from further analysis.

**Table 1:** Quality evaluation criteria. Four essential criteria were required for inclusion, indicating the study was internally valid. Studies meeting these four criteria were further evaluated on the sample, screening and outcome

Score	Quality Evaluation Criteria
**Essential internal validity**	
**✓**	**The sampling method and exclusion criteria did not introduce sampling bias.**
**✓**	**The design or analysis allowed for comparability between parameter and minimized co-intervention bias.**
**✓**	**The outcomes were reliable (e.g., all infants screened were accounted for; there was no error in the result tallies).**
**✓**	**The outcomes were valid (e.g., based on objective measures; did not contain bias).**
Sample	
*	The community was described from which all infants were drawn.
-	The community was not described from which infants were drawn.
*	The sample size was 1000 or more for each group described.
-	The sample size was less than 1000 for each group described.
*	The coverage rate was described and was 95% or more, as recommended by the Joint Committee of Infant Hearing.
-	The coverage rate was not described, or if described, coverage was less than 95%.
*	The infants included were described with respect to risk factors, NICU admission, well babies, all babies born, etc.
-	The infants included were not described.
Screening	
*	The screening protocol was described and included at least four of the five following factors: infant age at screening, test method, number of screens performed in screening step 1, the test device, and referral criteria (If not automatic) including one or both ears.
-	Less than four of the five above-mentioned factors pertaining to the screening protocol were described.
Outcome	
*	The method for collecting data was described (e.g., paper records, centralized database, etc).
-	The method for collecting data was not described.
*	The criteria for determining loss to follow-up (or nonattendance) was described^a^.
-	The criteria for determining loss to follow-up (or nonattendance) was not described^a^.

^a^Only relevant for studies reporting on loss to follow-up or nonattendance

The remaining studies were evaluated across three categories: the sample, screening, and outcome. One point was awarded for fulfilment of each of the criteria. This review reports on two outcomes (referral rate and LTFU from screening step 1) and different methods are used to assess these outcomes. The last criterion was thus only relevant for studies reporting on the outcome LTFU.

### Synthesis of included studies

The programme determinants investigated in each study were extracted. Studies were organised according to the programme determinant(s) and outcomes they investigated: referral rate, LTFU rate, or both The outcome of referral rate was derived based on the percentage or number of infants that failed and referred from screening step 1, out of the total number screened. LTFU rate was derived using the terminology provided in each study. There was some variation in the terminology used (e.g., the percentage of infants that did not attend follow-up, that defaulted, or that dropped out, out of the total number of infants expected to attend after a referral from screening step 1). For studies where sufficient data were provided for calculations, risk ratios with 95% confidence intervals and chi-square analyses were performed in SPSS v. 26. Chi-square analyses determine if differences reached statistical significance (*p* < 0.05). Risk ratios quantify the increased or decreased risk for referral or LTFU. Error bars that cross the axis at 1.0 indicate no significant difference between groups.

## Results

**Fig. 2:**
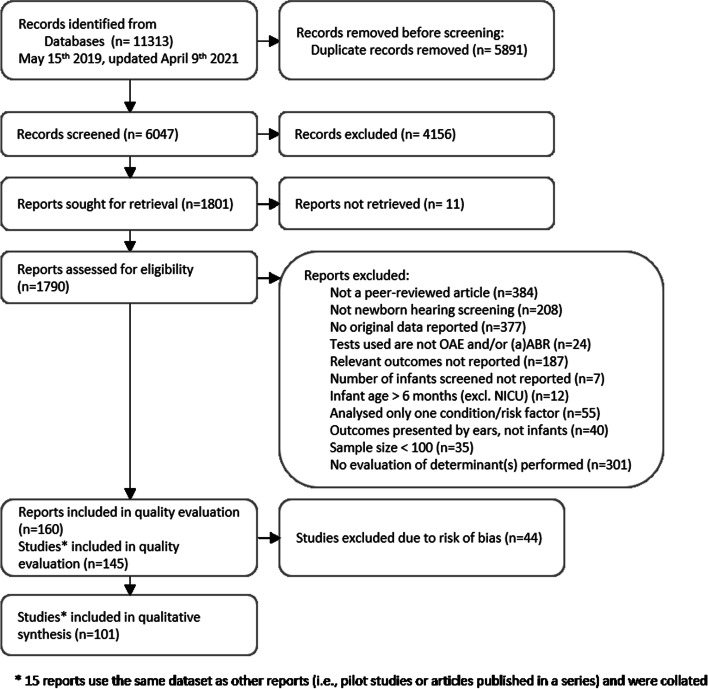
PRISMA (2020) flow chart

The results of the literature search and exclusions are displayed in Fig. 2. Non-English reports were excluded in the title and abstract review. There was a total of 905 non-English reports, out of which 359 would have otherwise met the title/abstract inclusion criteria. Full-text translations were not performed due to a lack of resources for translating the large number of publication languages among these reports (25 languages).

Out of the 1801 reports included in full text review, 11 could not be located by the Karolinska Institute Library in either virtual or paper form. The remaining reports were retrieved and reviewed. Excluded reports were sorted into categories based on the pre-determined criteria (Fig. 2). From the remaining 160 candidate reports, 10 included data that were used in larger or later published studies. Three reports from the *New York State universal newborn hearing screening demonstration* project were collated to one study, and four reports from the *Identification of neonatal hearing impairment* project were also collated to one study.

### Quality evaluation

A total of 145 studies were evaluated on methodological quality. Forty-four studies did not fulfil all four essential criteria (Table 1) and were excluded. A total of 101 studies were assessed further for quality and organised by outcome (referral rate or LTFU rate from screening step 1). All studies except two were observational cohort studies. The other two were non-randomised controlled trials [12, 13]. Fifty-eight studies reported only on referral rate, 35 investigated both the referral rate and LTFU from screening step 1, and eight reported only LTFU from screening step 1. Results of the quality evaluation for each article are available in Additional file 2 and Tables 3, 4, 5 and 6. Table 2 describes the percentage of studies fulfilling each of the quality criteria. For referral rate, only 8% of studies fulfilled all six criteria, 14% fulfilled five criteria, 33% fulfilled four criteria, 30% fulfilled 3 criteria, 11% fulfilled two criteria, and 4% fulfilled only one criterion. For LTFU rate, 5% percent of studies fulfilled all seven criteria, 12% fulfilled six criteria, 28% fulfilled five criteria, 37% fulfilled four criteria, 7% fulfilled three criteria, 12% fulfilled two criteria, and no studies fulfilled only one criterion.

**Table 2:** Summary of the quality evaluation regarding seven criteria for the two groups of studies evaluating the outcomes referral rate and/or loss to follow-up rate from screening step 1

Quality evaluation criteria	Percent of studies fulfilling criteria	
	Referral rate	Loss to follow-up rate
The community was described from which all infants were drawn.	95%	93%
The sample size was 1000 or more for each group described.	44%	60%
The coverage rate was described and was ≥95%, as recommended by the Joint Committee of Infant Hearing.	16%	30%
The infants included were described with respect to risk factors, NICU admission, well babies, all babies born, etc.	96%	95%
The screening protocol was described and included at least four of the five following factors: infant age at screening, test method, number of screens performed in screening step 1, the test device, and referral criteria (If not automatic) including one or both ears.	72%	58%
The method for collecting data was described (e.g., paper records, centralized database, etc).	44%	53%
The criteria for determining loss to follow-up (or nonattendance) was described.	Not relevant	35%

### Synthesis of programme determinants

A summary of studies and their findings are listed in Additional file 3. The following sections provide an overview of results. The first sections synthesise studies that described the association between referral rate from screening step 1 and protocol and programme determinants, starting first with screening method and then with other determinants. The subsequent section describes LTFU rate and the effect of programme determinants. Finally, the trends between infant group across both referral rate and LTFU are reported.

#### Referral rate and screening method

A total of 22 studies reported referral rates across different screening methods. A detailed description of studies is available in Additional file 4. Two studies compared TEOAE with distortion-product or tone-burst OAE [14, 15]. Sixteen studies compared TEOAE with aABR, one of which compared screening method only for NICU babies [16] and another only babies with RF [17]. The remaining 14 studies compared referral rates for WB only, WB and NICU babies independently, or all babies combined. These 14 studies are listed in Table 3. Studies either compared screening methods across two groups (between-subject) or using both methods on the same infant (within-subject). All studies used automatic OAE passing criteria. Most studies used 35 dB nHL passing criteria for aABR with a few exceptions, the Identification of Neonatal HI studies [18–21] (30 dB nHL), Konukseven et al. [22] (40 dB nHL) and Korres et al. [23] (not described). Risk ratios and 95% confidence intervals were calculated when data were available. For most within-subject studies, the data were not provided in the article to calculate risk ratios and confidence intervals.

**Table 3:**
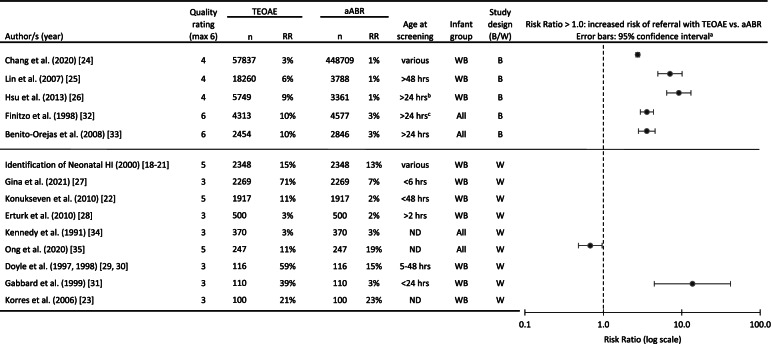
Referral rates from screening step 1 for studies that compared TEOAE with aABR screening for well babies [18–31] or all infants combined [32–35]. Studies are grouped by study design and ordered according to sample size

*ND* not described, *n* sample size, *RR* referral rate, *WB* well baby, *B* between-subject design, *W* within-subject design

^a^Risk ratios and confidence intervals for paired data (i.e., within-subject studies) could only be calculated for two studies that had reported data for infants who referred with both TEOAE and aABR, TEOAE but not aABR, and aABR but not TEOAE. Error bars that cross the axis at 1.0 indicate no significant difference

^b^24-h minimum for aABR and 48-h minimum for OAE. †24 -h minimum for vaginal births and 48-h minimum for cesarean section

In all between-subject studies, the use of aABR resulted in significantly lower referral rates from screening step 1 compared to TEOAE; however, heterogeneity across studies was observed between the within-subject studies that compared the two methods. In the two within-subject studies that showed the largest difference between methods [27, 31] screening was performed within 24 h from birth. Although most studies showed lower referral rates with aABR compared to TEOAE, the screening method is not the only solution to reducing referral rate, as indicated by the range of referral rates across studies, both for OAE (3 to 71%) and aABR (1 to 23%).

A two-technology versus single-technology screening protocol in step 1 was investigated by five studies (as described in Additional file 4). In a two-technology protocol, infants that fail OAE undergo aABR in the same screening step. In the comparison group the single-technology screening was performed twice before discharge in four out of five studies. All studies reported significantly lower referral rates with two-technology compared to single-technology screening with TEOAE [25, 32, 36–39]. Lin et al. [25] reported a lower referral rate with single-technology aABR (0.8%) compared to a two-technology protocol (2%), though Finitzo et al. [32] did not show a difference between methods (3%).

#### Referral rate and other programme determinants

Table 4 provides an overview of all programme determinants, aside from screening method, for the studies reporting on referral rate from screening step 1. Additional file 5 describes the findings for each programme determinant which is summarized in the following paragraphs. Overall, the synthesis showed that, in addition to screening method, an assortment of programme determinants influenced referral rates, such as the rescreening protocol, infant age and status, screening professional, and organization of the programme.

**Table 4:** Quality ratings for each study reporting on referral rate from screening step 1, grouped by the programme determinant studied

Programme determinant studied	Author/s (year) [citation]	Quality rating (/6)
Devices	Chan et al. (2015) [40]^a^	3
	Govaerts et al. (2001) [41]	4
	Murray et al. (2004) [42]^a^	3
	Deniz et al. (2020) [43]^a^	3
	Kishino et al. (2021) [44]^a^	3
Passing criteria	Akinpelu et al. (2019) [45]	4
	De Ceulaer et al. (1999), De Ceulaer et al. (2001) [46, 47]	3
	Gabbard et al. (1999) [31]	3
	Korres et al. (2003) [48]	4
	Korres et al. (2005) [49]	4
	Identification of Neonatal HI (Norton et al., 2000a, Norton et al., 2000b, Norton et al., 2000c, Sininger et al., 2000^a^) [18–21]	5
Rescreening within step 1	Burdzgla et al. (2007) [50]	1
	Clemens and Davis (2001) [51]^a^	6
	Korres et al. (2005) [49]	4
	Pastorino et al. (2005) [52]	3
	Shoup et al. (2005) [53]^a^	6
	Vernier et al. (2021) [54]	3
Infant status	Korres et al. (2005) [49]	4
	Vohr et al. (1993) [55]	1
Infant age since birth (inpatient)	Arslan et al. (2013) [56]	4
	Berninger and Westling (2011) [57]	5
	Chung et al. (2019) [58]^a^	4
	Dimitriou et al. (2016) [59]	3
	Hrncic et al. (2019) [60]	4
	Kelly et al. (2021) [61]^a^	4
	Korres et al. (2003) [62]	2
	Labaeka et al. (2018) [63]^a^	3
	Tabrizi et al. (2017) [64]	2
	Vernier et al. (2021) [54]	3
	Vohr et al. (1993) [55]	1
	Wessex Universal Neonatal Hearing Screening Trial Group (1998) [13]	4
Infant age / Inpatient vs. outpatient	Kanji et al. (2018) [65]	2
	Kolski et al. (2007) [66]	3
	Olusanya et al. (2009) [67]	4
	Scheepers et al. (2014) [68]	4
	Uilenburg et al. (2009) [69]	3
Screening professional and experience	de Kock et al. (2016) [70]^b^	5
	Gallus et al. (2020) [71]	3
	Stewart et al. (2000) [72]^a^	3
Hospital size and setting for step 1	Fan et al. (2010) [73]^a^	3
	Grasso et al. (2008) [74]	4
	Hergils (2007) [75]	6
	Mehl and Thomson (2002) [76]^a^	4
	Olusanya (2010) [77]	5
	Scheepers et al. (2014) [68]	4
Programme organisation	Barker et al. (2013) [78]^a^	5
	Park et al. (2020) [79]^b^	3

^a^aABR used

^b^aABR and OAE used

For studies investigating devices for screening, results can be found in Additional file 5. For passing criteria, OAE signal-to-noise ratio (SNR) passing criteria of 3 versus 6 dB did not result in a significant decrease in a referral rate, shown across multiple studies [31, 46, 48, 49]. The one study that compared aABR passing intensity reported that, out of the infants that failed screening at 30 dB nHL, 60% would have passed if screening was instead performed at 50 dB nHL [21]. When rescreening is performed immediately after a failed screening attempt or just before discharge from the maternity hospital, referral rates from step 1 are reduced for both OAE and aABR screening [49–52].

**Fig. 3:**
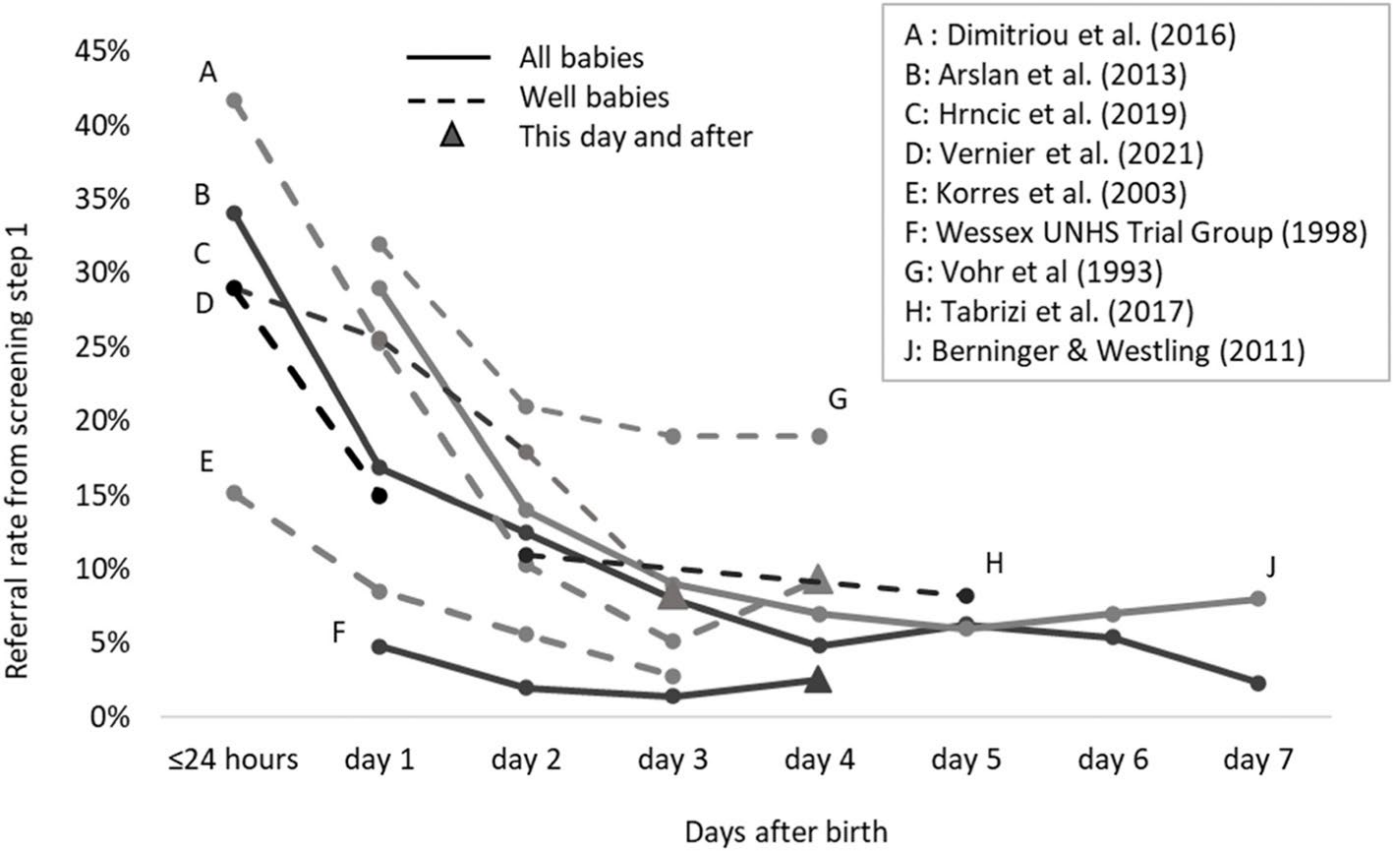
Age when screened with TEOAE and referral rate from step 1

With regards to infant-level factors and referral rates, timing is important. Screening when infants are quiet and/or sleeping) significantly reduced referral rate [49, 55]. The age of the infant when screened also influenced referral rates. A clear trend was found between day 0 and day 3, but studies reported varying results after day 3. Figure 3 displays the referral rates by age of the infant when screened for studies using OAE on all babies or WB across nine studies [13, 55–57, 59, 60, 62, 64]. Some studies continued to show a reduction in referral rates after day 3 to 5. Four studies reported an increase in referral rate [13, 56, 57, 59], three of which reported referral rates for all babies, including NICU babies [13, 56, 57]. For aABR, a single-centre study found a reduction in referral rate from 0 to 8 h (22%) to 39–48 h after birth (11%). Chung et al. [58] showed small differences from day 0 to 7 (0.4 to 1.5%) in a multi-site study.

After the first few days from birth, referral rates may remain somewhat stable up to a few weeks of age [69]. According to two studies, once infants were a couple months of age, referral rates were higher [58, 66]. In Chung et al., aABR referral rates were lowest within the first week after birth and increased from 1.5 to 4.7% from 1 week to 2 months of age [58]. Another study that compared OAE referral rates showed a higher rate of bilateral referrals at 2 months of age (3%) at an outpatient clinic compared to just prior to discharge in the maternity hospital (1%) [66]; however, it is difficult to limit this interpretation only to age, as the location of screening also differed.

The synthesis of results showed some ambiguity with regards to screener- and organization-level determinants on referral rates. A more experienced screener had lower referral rates compared to a less experienced screener according to one study, but only with OAE and not with aABR [70]. Another study showed no learning curve for OAE in four groups of screeners across a 12-month period [71]. Two studies showed that hospitals with more annual births were more successful at achieving low referral rates [75, 76]. Conversely, a third study found that a hospital with high birth rates had higher referral rates due to the increased burden and stress to screen more infants before discharge, which resulted in infants being screened earlier [68]. The remaining two studies showed negligible referral rate differences between larger and smaller hospitals, possibly due to the existence of networks and collaboration between sites [73, 75]. Finally, for programme organisation, both studies showed lower referral rates when NHS was embedded into a larger universal NHS programme [78, 79].

#### Loss to follow-up rate and programme determinants

Table 5 lists the programme determinants and studies reporting on the outcome LTFU from step 1. Additional file 5 describes the findings for each programme determinant which are summarized in the following text. For passing criteria or rescreening in step 1, comparisons did not show reliable trends for improving LTFU [46–49]. The factors that influenced LTFU included screeners, hospital size, and the organization of the programme.

**Table 5:** Quality ratings for each study reporting on loss to follow-up rate (LTFU) from screening step 1, grouped by the programme determinant studied

Programme determinant studied	Author/s (year) [citation]	Quality rating (/7)
Passing criteria	De Ceulaer et al. (1999), De Ceulaer et al. (2001) [46, 47]	3
	Korres et al. (2003) [48]	5
	Korres et al. (2005) [49]	4
Rescreening within step 1	Korres et al. (2005) [49]	4
Infant status	Korres et al. (2005) [49]	4
Infant age / Inpatient vs. outpatient	Kolski et al. (2007) [66]	3
	Scheepers et al. (2014) [68]	4
	Uilenburg et al. (2009) [69]	4
Screening professional and experience	Cunningham et al. (2018) [80]	5
	de Kock et al. (2016) [70]	6
	Thomson and Yoshinaga-Itano (2018) [81]	5
Screener training	Cunningham et al. (2018) [80]	5
Audiologist involvement	Cunningham et al. (2018) [80]	5
	Thomson and Yoshinaga-Itano (2018) [81]	5
Hospital size and setting for step 1	Mehl and Thomson (2002) [76]	5
	Scheepers et al. (2014) [68]	4
	Thomson and Yoshinaga-Itano (2018) [81]	5
Programme organisation	Barker et al. (2013) [78]	6
	Park et al. (2020) [79]	4
Step 2 scheduling and fees	Cunningham et al. (2018) [80]	5
	Thomson and Yoshinaga-Itano (2018) [81]	5
Compliance with guidelines	Cunningham et al. (2018) [80]	5
Step 1 referral rate	Thomson and Yoshinaga-Itano (2018) [81]	5
Step 2 location	Barker et al. (2013) [78]	6
	Cunningham et al. (2018) [80]	5
	Hunter et al. (2016) [12]	5
	Thomson and Yoshinaga-Itano (2018) [81]	5
	Uilenburg et al. (2009) [69]	4

For screener-level determinants, Thomson and Yoshinaga-Itano found that LTFU rates were lower for hospitals that had technicians as screeners compared to hospitals with nurses and volunteers as screeners [81]. However, there were no differences between screener professional if an audiologist was involved in the hospital NHS programme. In fact, audiologist involvement was the most influential factor for LTFU after step 1 referral. Once audiologist involvement was incorporated in the regression model, many other programme factors became non-significant. The significance of audiologist involvement was not observed in the data collected later by Cunningham et al. [80] in the same region. This apparent discrepancy between studies was attributed to the increase in resources for adding audiologist involvement to hospitals since findings were initially produced by Thomson and Yoshinaga-Itano [81]. In Cunningham et al., only seven hospitals out of 53 reported no audiologist involvement [80].

Bigger hospitals with more annual births had lower LTFU rates, compared to smaller hospitals [76, 81, 82]. One study was the exception [68]. They showed a reverse trend, likely due to the increased workload and stress on screeners from the busier hospital. Thomson and Yoshinaga Itano reported that hospitals with birth rates from 2000 to 3000 per year had the highest LTFU, compared to both larger and smaller hospitals [81]. However, these were also the hospitals that had more volunteer screeners, and parents were mostly responsible for scheduling the step 2 appointment.

With regards to NHS organization, LTFU rates were lower when local NHS was embedded in a larger (regional or national) universal NHS programme [78, 79]. If parents are responsible for scheduling step 2, LTFU are higher, except when audiology involvement is incorporated into the analysis [81]. Implementing a fee for step 2 screening was also associated with higher LTFU [80]. Additionally, adherence to hospital NHS guidelines was not associated with LTFU rate [80], nor was referral rate from step 1 (once audiologist involvement was considered) [81].

The location of step 2 screening was related to LTFU. A slightly higher LTFU rate was observed when step 1 and step 2 screening was performed in a well-baby clinic compared to at home [69]. LTFU was higher if families were referred to an outpatient clinic for follow-up, compared to if families were asked to return to the screening hospital for step 2 [78, 81]. Hunter et al. showed that performing step 2 in a collaborating outpatient centre that is accessible to low-income families reduced overall LTFU [12].

#### Referral and loss to follow-up rate and infant group

**Fig. 4:**
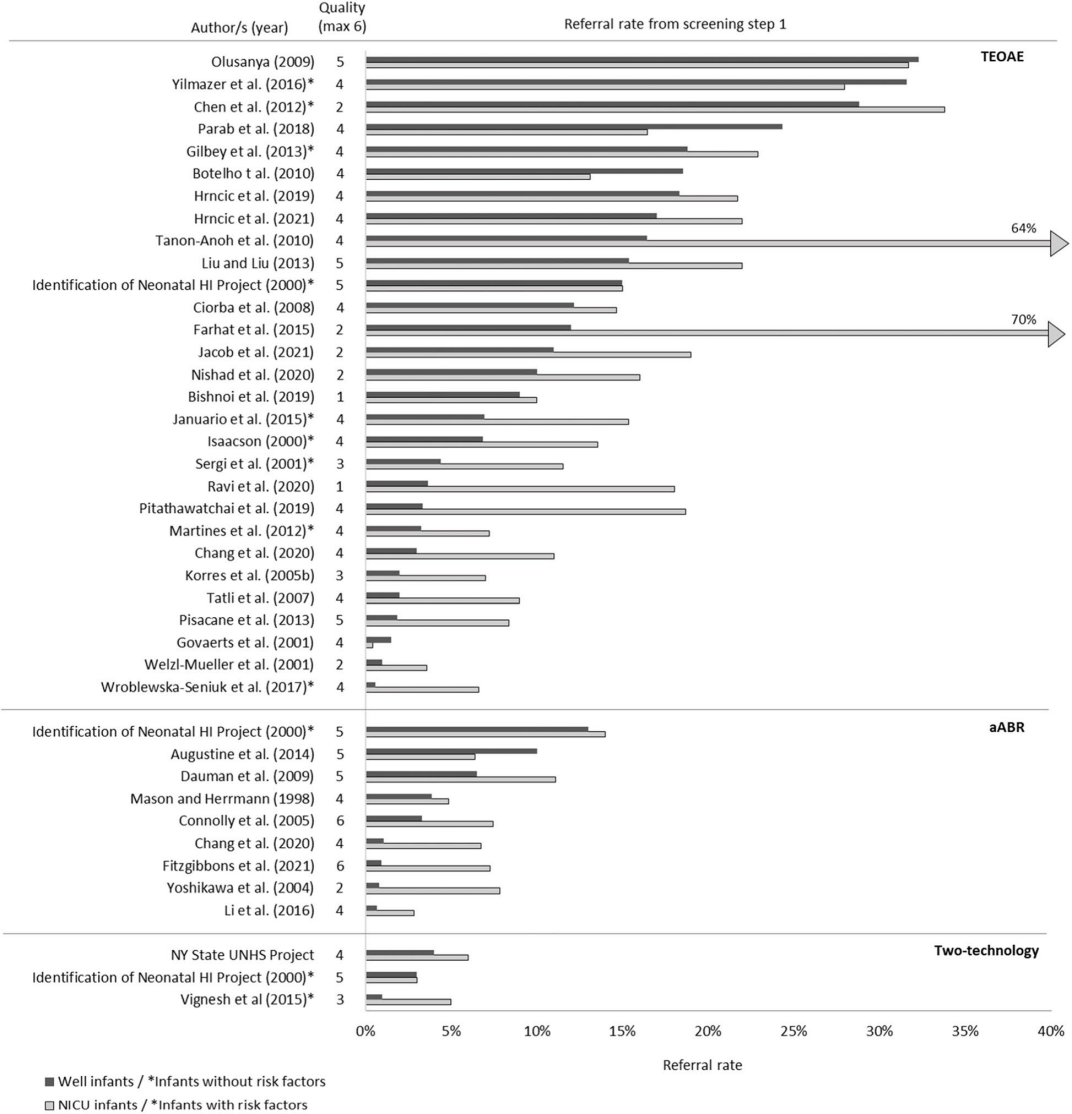
Referral rates from step 1 for studies comparing well babies / babies without risk factors to NICU babies / babies with risk factors

A total of 38 studies reported on referral rates between infant groups, displayed in Fig. 4 grouped by screening method. In most studies, referral rates were higher for NICU babies or babies with RF, compared to WB babies or babies without RF [24, 36, 60, 83–115]. Some studies were exceptions where the trend between infant groups was not as clear [41, 67, 116–119], particularly among studies with high overall step 1 referral rates. Figure 4 also demonstrates the large range in referral rates across studies.

For LTFU rate, the trend between infant groups is not consistent across studies. Twenty-three out of twenty-four studies are displayed in Table 6. A risk ratio > 1.0 indicate a higher risk of LTFU for NICU babies or babies with RF, compared to WB or babies without RF. Error bars that cross the axis at 1.0 indicate no significant differences between groups. Studies were sorted according to LTFU rates for WB. A quantitative analysis was not performed due to the heterogeneity across studies. For studies where LTFU was low in the WB population (< 20%), a more consistent trend is observed where NICU / RF babies were more at risk for LTFU. The duration between steps 1 and 2 were also shorter in these programmes. For the studies reporting high LTFU for WB (over 20%), results across studies were more variable.

**Table 6:**
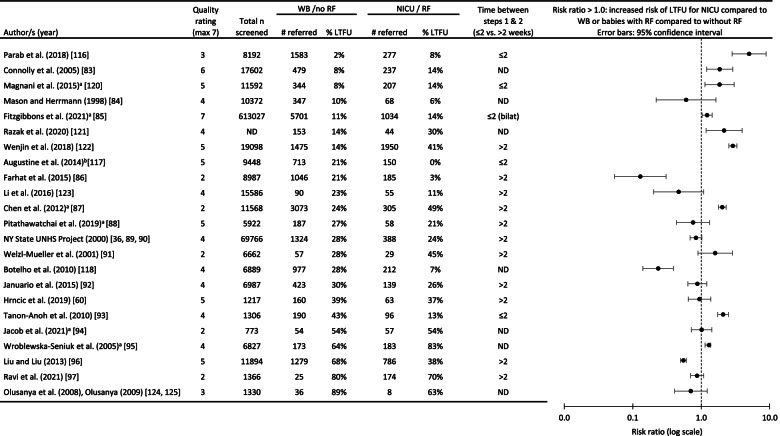
Percentage of children lost to follow-up (LTFU) out of those referred from screening step 1, for the studies comparing LTFU rates between infant groups [36, 60, 83–97, 116–118, 120–125]

*LTFU* lost to follow-up, *n* sample size, *ND* not described, *NICU* neonatal intensive-care unit, *RF* risk factors, *WB* well baby

^a^Studies that compared infants with risk factors for permanent hearing impairment compared to infants without risk factors. Other studies compared WB with infants admitted to the NICU

^b^Risk ratio cannot be calculated with a LTFU rate of 0%

Two studies investigated infant group differences in a multivariate logistic regression and reported corrected odds ratios, thus controlling for other infant and family-level factors. Vohr et al. [126] was not included in Table 5 as group LTFU rates were not available. They reported that NICU infants were almost 6 times more likely to be LTFU than WB (95% CI of 3.55 to 9.98). More recently, Razak et al. [121] reported that infants with an extended NICU stay (> 5 days) were 2.3 times more likely to be LTFU (95% CI 1.03–5.30) compared to infants with NICU stay of 0 to 5 days.

## Discussion

Systematically high referral and LTFU rates reduce the effectiveness of NHS programmes, as more infants with normal hearing are referred and fewer infants with PHI are detected. This systematic review identified 101 studies that compared referral rate and/or LTFU rate from step 1 between protocols, programme factors or infant risk groups. The reported referral and the LTFU rates were very diverse, reflecting a range of effectiveness across NHS programmes. No one determinant was shown to be a single solution to achieving optimal referral or LTFU rates. For instance, most studies reported lower referral rates for aABR compared to TEOAE for WB; however, simply switching from OAE to aABR does not appear to be sufficient for reducing referral rate to very low levels for all programmes. Similarly, the heterogeneity across studies on LTFU signifies its complexity. Expert groups and policymakers may need to consider the combination of various determinants that can be most successful in improving the overall quality of their programme, given the local context and available resources. The results of this review describe key determinants that can be considered.

Referral rates with OAE were consistently lower if screening was performed at 3 days of age or later, compared to screening performed before day 3. Many existing NHS protocols recommend waiting at least 24 to 48 h after birth before attempting step 1 screening to avoid unnecessary referrals [6]. However, as maternity ward stays are shortening for WB across many countries [127], protocols may need to be revised to accommodate this trend. This may include revising the step 1 protocol by switching from TEOAE to aABR for all infants, adding a rescreen before discharge (either with the same technology or with aABR after a failed OAE), or by moving step 1 screen to an outpatient setting. Moving step 1 to an outpatient setting can improve referral rates if inpatient screening is performed only hours after birth [65]; however, policymakers must also consider whether these factors could affect the sensitivity of the screening programme and whether coverage rates will be negatively affected by such a reorganisation [66].

Replacing TEOAE screening with aABR for WB can reduce step 1 referral rates. Out of the 14 studies comparing aABR and TEOAE, all except two had lower referral rates for aABR screening. Gina et al. [27] showed the largest difference in rates between methods (71% versus 13%), when testing was performed before 6 h of age. However, other studies showed more negligible differences, and one study even revealed significantly higher referral rates for aABR [35]. They attributed their unique finding to the years of prior experience screeners had with OAE compared to aABR, which was newly trained and practiced prior to commencement of the study. It was unclear from the studies in this systematic review if practice alone improves referral rate [71, 75, 76]: though, part of improving the quality of the programme could involve other aspects related to experience, such as planning the optimal time for OAE screening to accommodate the behaviour of the infant [49, 55], networking with larger hospitals [75], or embedding smaller NHS programmes into a larger programme [78, 79]. If aABR screening is being considered as the primary screening method for WB, policymakers should also consider the challenges associated with a new technology and consider the advantages and disadvantages in relation to the current quality of their programme. For example, the detection of auditory neuropathy and reduction of referral rate should be weighted relative to the added cost. Furthermore, aABR screening using passing intensities from 35 to 45 dB nHL will also miss mild to moderate hearing impairment [128, 129], which might otherwise be detected with TEOAE.

Two influential programme-level factors for LTFU, as evaluated through this systematic review, were NHS personnel and organisation. Specifically, the involvement of an audiologist (or perhaps another expert in audiology) was a key factor for achieving low LTFU rates, according to Thomson and Yoshinaga-Itano [81]. When this factor was incorporated into the logistic regression model, the effect of other programme-level factors decreased. For instance, audiologist involvement reduced the impact of hospital size, which was otherwise shown to be an influential factor in other studies, with larger hospitals having lower LTFU rates compared to smaller hospitals [76, 81, 82]. Because of the multifaceted barriers that cause LTFU, an audiologist involved in the programme can monitor the performance outcomes of the screening and focus improvement plans within the local context. Across countries, various health professionals are responsible for performing screening, such as nurses, technicians, ENT surgeons, physicians and audiologists [6]. Thompson and Yoshinaga-Itano [81] examined a U.S. based NHS programme where nurses or technicians perform screening, overseen by an audiologist. Although these findings are only reported in one study, the quality of this study is high. It is unclear, however, whether these findings would extrapolate to countries outside the U.S. that have different health care systems and different training programmes for screening staff and audiologists.

One improvement plan for LTFU may be restructuring the NHS programme, such as incorporating smaller area / hospital-based programmes into a regional or national tracking system [79] or determining the optimal location and timing for step 2 [12] based on the culture, postnatal care structure, and resources of the local population. Timely scheduling and accessibility to step 2 screening can have a positive impact on families returning for follow-up [12]; however, accessibility may vary depending on the dynamics of the population. For example, infants admitted to NICU may have been transferred from their original birth hospital and therefore have further to travel to return for step 2. In this review, NICU infants tended to have a higher risk for LTFU compared to WB, particularly if the programme reported low LTFU for WB, although the trend was not homogeneous across studies. This trend is also not surprising considering that a more serious health condition may take priority over hearing impairment in terms of time, attention and associated costs. Studies with poorer LTFU for WB did not show a pattern between infant groups, indicating the likely involvement of other factors that are more strongly associated with high LTFU.

In this review, 35 studies reported on both referral and LTFU rate outcomes, 58 reported on referral rate only, and eight reported on LTFU rate only. LTFU after NHS referral is a significant problem for successfully detecting children with PHI in many countries [5]. The fact that population-based studies on LTFU are lacking in comparison to referral rate may be due to how NHS data monitoring and evaluation are performed. In our survey of 42 NHS programmes, only 12 could report valid data on follow-up [5]. In many countries and regions, quality evaluation of NHS performance may end at the level of screening referral, leaving the number of infants LTFU undocumented. Without monitoring the number of infants LTFU, it is impossible to assess whether this indicator requires improvement and the optimal strategies to manage it.

A lack of reporting was also evident in the quality evaluation. A lack of detail is not surprising, as it has been discussed in other studies on early detection and intervention of hearing impairment [130]. Though, it is particularly remarkable that of the studies that investigated the outcome LTFU, only 35% described how LTFU was defined. LTFU can be defined in different ways. For example, it could be interpreted as the percentage of all referred infants who do not attend the scheduled follow-up appointment, it could exclude infants who relocated or sought follow-up elsewhere, or it could include only infants whose families refused follow-up or could not be contacted via telephone. Because the bulk of studies did not provide a definition, this limits the generalisability of the influence of programme factors on this outcome.

It is important to note that, in this review, over 900 records were excluded because reports were not written English, and out of these, a large number (359 reports) would have otherwise fulfilled the title / abstract inclusion criteria. It is unknown whether any of these non-English reports would have been eligible for inclusion after the full-text review. Given that this study aimed to investigate the outcomes from NHS programmes using various protocol and programme factors, information published in languages aside from English could potentially add valuable information to the findings. Despite this possible language bias, the results of this review included studies from 31 countries across six continents.

Given the diversity of settings, it is important that policymakers consider the local context, in addition to the programme-level factors identified in this review. For instance, in contexts or settings where LTFU is problematic, reducing the referral rate from step 1 may be particularly beneficial, as fewer infants referred means that fewer infants are LTFU. There are multiple frameworks for performing quality improvement. A common start is identifying the problem and formulating possible solutions [131]. This systematic review provides an overview from the literature of the reported solutions for improving NHS effectiveness using various protocol and programme modifications. However, not included in this review is the dynamics of the local context. Organisational- and macro-level factors, such as existing peri-and postnatal care practice, national policies, funding and governance should all be evaluated with respect to implementation and quality improvement. Demographic and cultural characteristics of the infants and their families (e.g., ethnicity, insurance plans, education and distance to the hospital) [80] and acceptability of screening [132] may all be associated with NHS outcomes and should also be addressed relative to implementation and a quality improvement plan for a local NHS programme.

## Conclusion

This systematic review identified key protocol and programme level factors that can influence referral rate and LTFU from step 1. For most studies, referral rates were lower for aABR compared to TEOAE. Referral rates were also lower for two-technology screening (OAE, aABR) when compared to single-technology TEOAE screening performed twice. Other programme factors that influenced referral rate included rescreening within step 1, the age of the infant when screened, and screening experience. Programme factors that influenced LTFU rate were the screener, audiologist involvement, and the organization of NHS including the selected location for step 2. In summary, no single determinant was identified to reduce referral or LTFU rates. The range of referral and LTFU rates across studies and their heterogeneity justifies the need for expert groups and policymakers to evaluate possible solutions to improving quality based on their local context.

## Supplementary Information

**Additional file 1.** Search Strategy used in Ovid Medline.

**Additional file 2.** Results of quality evaluation.

**Additional file 3.** Overview of all studies included in the systematic review.

**Additional file 4.** Studies comparing referral rates across screening methods.

**Additional file 5.** Summary of findings for programme determinants, divided into protocol-, individual- and organizational-levels.

## Data Availability

Not applicable.

## Abbreviations

aABR: Automated auditory brainstem response
DPOAE: Distortion-product otoacoustic emissions
LTFU: Loss to follow-up
NHS: Newborn hearing screening
NICU: Neonatal intensive-care unit
OAE: Otoacoustic emissions
PHI: Permanent hearing impairment
RF: Risk factors
TEOAE: Transient-evoked otoacoustic emissions
WB: Well baby
