# Supplementary material for: Protocol and programme factors associated with referral and loss to follow-up from newborn hearing screening: a systematic review
Source: BMC Pediatr. 2022 Aug 5;22:473. doi: 10.1186/s12887-022-03218-0 (PMC9354382; doi:10.1186/s12887-022-03218-0)
Supplement: Supplementary file 1 — Additional file 1. Search Strategy used in Ovid Medline. [file 12887_2022_3218_MOESM1_ESM.docx]

Additional File 1: Search Strategy used in Ovid Medline

The search strategy was adapted for each database searched. The following search strategy is the example used to search Ovid Medline. Appropriate modifications were used for searching Embase, Cochrane Library, Web of Science Core Collection and Cinahl.

**Field labels**

- exp/ = exploded MeSH term
- / = non exploded MeSH term
- .ti,ab,kf. = title, abstract and author keywords
- adjx = within x words, regardless of order
- * = truncation of word for alternate endings

| 1 | exp Hearing/ |
| --- | --- |
| 2 | Hearing disorders/ |
| 3 | exp Hearing loss/ |
| 4 | exp Evoked Potentials, Auditory/ |
| 5 | (hearing* or deaf* or PCHI or PCHL or hypoacus* or hypacus*).ti,ab,kf. |
| 6 | (ABR or ABRs or aABR* or a-ABR* or auditory evoked or BERA or otoacoustic emission* or oto-acoustic emission* or transient evok* or distortion-product* or click-evok* or OAE* or TEOAE* or DPOAE* or COAE* or EOAE* or AOAE* or a-OAE* or TOAE*).ti,ab,kf. |
| 7 | (brainstem adj3 (auditory or audiometr*) adj3 (response* or potential*)).ti,ab,kf. |
| 8 | or/1-7 |
| 9 | Neonatal screening/ |
| 10 | Mass screening/ |
| 11 | ((audio* or auditory or deaf* or hearing) adj4 (screen* or program*)).ti,ab,kf. |
| 12 | ((ABR or ABRs or aABR* or a-ABR* or auditory evoked or BERA or otoacoustic emission* or oto-acoustic emission* or transient evok* or distortion-product* or click-evok* or OAE* or TEOAE* or DPOAE* or COAE* or EOAE* or AOAE* or a-OAE* or TOAE*) adj4 (screen* or program*)).ti,ab,kf. |
| 13 | (brainstem adj3 (auditory or audiometr*) adj3 (response* or potential*) adj4 (screen* or program*)).ti,ab,kf. |
| 14 | or/9-13 |
| 15 | exp Infant/ |
| 16 | Nurseries/ |
| 17 | Intensive Care Units, Neonatal/ |
| 18 | (infant or infants or infancy or newborn* or neonat* or baby or babies or maternity or nurser* or NICU*).ti,ab,kf. |
| 19 | or/15-18 |
| 20 | 8 and 14 and 19 |
