# Supplementary material for: Protocol and programme factors associated with referral and loss to follow-up from newborn hearing screening: a systematic review
Source: BMC Pediatr. 2022 Aug 5;22:473. doi: 10.1186/s12887-022-03218-0 (PMC9354382; doi:10.1186/s12887-022-03218-0)
Supplement: Supplementary file 2 — Additional file 2. Results of quality evaluation. [file 12887_2022_3218_MOESM2_ESM.docx]

Additional file 2: Results of quality evaluation

Studies that report on the referral rate (Table 1) and/or loss to follow-up rates (Table 2) as outcomes from screening step 1 were evaluated on quality. Studies that report on both outcomes are listed in both tables and indicated with a gray background. A star was awarded to studies meeting each quality evaluation criterion.

Criteria are:

1. The community was described from which all infants were drawn
2. The sample size was 1000 or more for each group described
3. The coverage rate was described and was 95% or more, as recommended by the Joint Committee of Infant Hearing.
4. The infants included were described with respect to risk factors, NICU admission, well babies, all babies born, etc.
5. The screening protocol was described and included at least four of the five following factors: infant age at screening, test method, number of screens performed in screening step 1, the test device, and referral criteria (If not automatic) including one or both ears.
6. The method for collecting data was described (e.g., paper records, centralized database, etc).
7. The criteria for determining loss to follow-up (or nonattendance) was described (Table 2 only)

**Table 1. Results of the quality evaluation for the included studies that measure the referral rate from screening step 1.**

| **Author/s (year)** | **Quality evaluation of studies that measure referral rate** | | | | | | |
| --- | --- | --- | --- | --- | --- | --- | --- |
|  | **(1) Community** | **(2) Sample**  **≥ 1000** | **(3) Coverage ≥95%** | **(4) Infant group** | **(5) Protocol** | **(6)**  **Data collection** | **#** |
| Akinpelu et al. (2019) | * |  |  | * | * | * | 4 |
| Arslan et al. (2013) | * |  |  | * | * | * | 4 |
| Augustine et al. (2014) | * | * | * | * | * |  | 5 |
| Barker et al. (2013) | * | * |  | * | * | * | 5 |
| Benito-Orejas et al. (2008) | * | * | * | * | * | * | 6 |
| Berninger and Westling (2011) | * | * | * |  | * | * | 5 |
| Bishnoi et al. (2019) |  |  |  | * |  |  | 1 |
| Botelho et al. (2010) | * | * |  | * |  | * | 4 |
| Burdzgla et al. (2007) |  |  |  | * |  |  | 1 |
| Chalkiadakis et al. (2014) | * |  |  | * | * |  | 3 |
| Chan et al. (2015) | * |  |  | * | * |  | 3 |
| Chang et al. (2020), Chung et al. (2019), Chung et al. (2020) | * | * |  | * |  | * | 4 |
| Chen et al. (2012) | * |  |  | * |  |  | 2 |
| Ciorba et al. (2008), Ciorba et al. (2007) | * | * |  | * |  | * | 4 |
| Clemens and Davis (2001) | * | * | * | * | * | * | 6 |
| Connolly et al. (2005) | * | * | * | * | * | * | 6 |
| Dauman et al. (2009) | * | * |  | * | * | * | 5 |
| De Ceulaer et al. (1999), (De Ceulaer et al., 2001) | * |  |  | * | * |  | 3 |
| de Kock et al. (2016) | * | * |  | * | * | * | 5 |
| Deniz et al. (2020) |  |  |  | * | * | * | 3 |
| Dimitriou et al. (2016) | * |  |  | * | * |  | 3 |
| Doyle et al. (1997), Doyle et al. (1998) | * |  |  | * | * |  | 3 |
| Erturk et al. (2010) | * |  |  | * | * |  | 3 |
| Fan et al. (2010) | * | * |  | * |  |  | 3 |
| Farhat et al. (2015) | * |  |  | * |  |  | 2 |
| Finitzo et al. (1998) | * | * | * | * | * | * | 6 |
| Fitzgibbons et al. (2021) | * | * | * | * | * | * | 6 |
| Gabbard et al. (1999) | * |  |  | * | * |  | 3 |
| Gallus et al. (2020) | * |  |  | * | * |  | 3 |
| Gilbey et al. (2013) | * |  | * | * |  | * | 4 |
| Gina et al. (2021) | * | * |  | * |  |  | 3 |
| Govaerts et al. (2001) | * |  |  | * | * | * | 4 |
| Grasso et al. (2008) | * | * |  | * | * |  | 4 |
| Hergils (2007) | * | * | * | * | * | * | 6 |
| Hrncic et al. (2019) | * |  |  | * | * | * | 4 |
| Hrncic et al. (2021) | * |  | * | * |  | * | 4 |
| Hsu et al. (2013) | * | * |  | * | * |  | 4 |
| Identification of neonatal hearing impairment project (Norton et al., 2000a, Norton et al., 2000b, Norton et al., 2000c, Sininger et al., 2000) | * | * |  | * | * | * | 5 |
| Isaacson (2000) | * |  |  | * | * | * | 4 |
| Jacob et al. (2021) | * |  |  | * |  |  | 2 |
| Januario et al. (2015) | * |  |  | * | * | * | 4 |
| Kanji et al. (2018) | * |  |  | * |  |  | 2 |
| Kelly et al. (2021) | * |  |  | * | * | * | 4 |
| Kennedy et al. (1991) | * |  |  | * | * |  | 3 |
| Kennedy et al. (2000) | * | * |  |  |  |  | 2 |
| Kishino et al. (2021) | * |  |  | * | * |  | 3 |
| Kolski et al. (2007) | * | * |  | * | * |  | 3 |
| Konukseven et al. (2010) | * | * |  | * | * | * | 5 |
| Korres et al. (2003a) | * | * |  | * | * |  | 4 |
| Korres et al. (2003b) | * |  |  | * | * |  | 2 |
| Korres et al. (2005a) | * | * |  | * | * |  | 4 |
| Korres et al. (2005b) | * | * |  | * | * |  | 3 |
| Korres et al. (2006) | * |  |  | * | * |  | 3 |
| Labaeka et al. (2018) | * |  |  | * | * |  | 3 |
| Li et al. (2016) | * | * | * | * |  |  | 4 |
| Lin et al. (2005), Lin et al. (2007) | * | * |  | * | * |  | 4 |
| Liu and Liu (2013) | * | * |  | * | * | * | 5 |
| Martines et al. (2007), Martines et al. (2012) | * |  |  | * | * | * | 4 |
| Mason and Herrmann (1998) | * | * |  | * | * |  | 4 |
| McPherson et al. (2006) | * |  |  | * | * |  | 3 |
| Mehl and Thomson (2002) | * | * |  | * |  | * | 4 |
| Meyer et al. (1999) | * |  |  | * | * |  | 3 |
| Murray et al. (2004) | * |  |  | * | * |  | 3 |
| New York State UNHS project (Gravel et al., 2000, Spivak et al., 2000, Prieve et al., 2000) | * | * |  | * | * |  | 4 |
| Nishad et al. (2020) |  |  |  | * | * |  | 2 |
| Olusanya et al. (2008), Olusanya (2009) | * |  | * | * | * | * | 5 |
| Olusanya (2010) | * | * |  | * | * | * | 5 |
| Olusanya et al. (2009) | * | * |  | * |  | * | 4 |
| Ong et al. (2020) | * |  |  | * | * | * | 5 |
| Parab et al. (2018) | * | * |  | * |  |  | 3 |
| Park et al. (2020) | * | * |  |  |  | * | 3 |
| Pastorino et al. (2005) | * |  |  | * | * |  | 3 |
| Pisacane et al. (2013) | * | * |  | * | * | * | 5 |
| Pitathawatchai et al. (2019) | * |  | * | * |  | * | 4 |
| Ravi et al. (2021) |  |  |  | * |  |  | 1 |
| Scheepers et al. (2014) | * |  |  | * | * | * | 4 |
| Sergi et al. (2001) | * |  |  | * | * |  | 3 |
| Shang et al. (2016) | * | * |  | * | * |  | 4 |
| Shoup et al. (2005) | * | * | * | * | * | * | 6 |
| Stewart et al. (2000) | * |  |  | * | * |  | 3 |
| Tabrizi et al. (2017) | * |  |  | * |  |  | 2 |
| Tanon-Anoh et al. (2010) | * |  |  | * | * | * | 4 |
| Tatli et al. (2007) | * |  |  | * | * | * | 4 |
| Tzanakakis et al. (2016) | * | * | * | * | * |  | 5 |
| Uilenburg et al. (2009) | * |  |  | * | * |  | 3 |
| Vernier et al. (2021) | * |  |  | * |  | * | 3 |
| Vignesh et al. (2015) | * |  |  | * | * |  | 3 |
| Vohr et al. (1993) | * |  |  |  |  |  | 1 |
| Welzl-Mueller et al. (2001) | * |  |  | * |  |  | 2 |
| Wessex Universal Neonatal Hearing Screening Trial Group (1998) | * | * |  | * | * |  | 4 |
| Wroblewska-Seniuk et al. (2005), Wroblewska-Seniuk et al. (2017) | * |  |  | * | * | * | 4 |
| Yilmazer et al. (2016) | * | * |  | * | * |  | 4 |
| Yoshikawa et al. (2004) | * |  |  | * |  |  | 2 |

**Table 2. Results of the quality evaluation for the included studies that measure the loss to follow-up rate after screening step 1.**

| **Author/s (year)** | **Quality evaluation for studies that measure loss to follow-up rate** | | | | | | | |
| --- | --- | --- | --- | --- | --- | --- | --- | --- |
|  | **(1) Community** | **(2) Sample**  **> 1000** | **(3) Coverage >95%** | **(4) Infant group** | **(5) Protocol** | **(6)**  **Data collection** | **(7) Follow-up** | # |
| Augustine et al. (2014) | * | * | * | * | * |  |  | 5 |
| Barker et al. (2013) | * | * |  | * | * | * | * | 6 |
| Benito-Orejas et al. (2008) | * | * | * | * | * | * |  | 6 |
| Botelho et al. (2010) | * | * |  | * |  | * |  | 4 |
| Chen et al. (2012) | * |  |  | * |  |  |  | 2 |
| Connolly et al. (2005) | * | * | * | * | * | * |  | 6 |
| Cunningham et al. (2018) | * | * |  | * |  | * | * | 5 |
| De Ceulaer et al. (1999) | * |  |  | * | * |  |  | 3 |
| de Kock et al. (2016) | * | * |  | * | * | * | * | 6 |
| Erturk et al. (2010) | * |  |  | * | * |  | * | 4 |
| Farhat et al. (2015) | * |  |  | * |  |  |  | 2 |
| Finitzo et al. (1998) | * | * | * | * | * | * |  | 6 |
| Fitzgibbons et al. (2021) | * | * | * | * | * | * | * | 7 |
| Hrncic et al. (2021) | * |  | * | * |  | * | * | 5 |
| Hunter et al. (2016) | * | * |  | * |  | * | * | 5 |
| Jacob et al. (2021) | * |  |  | * |  |  |  | 2 |
| Januario et al. (2015) | * |  |  | * | * | * |  | 4 |
| Kolski et al. (2007) |  | * |  | * | * |  |  | 3 |
| Korres et al. (2003a) | * | * |  | * | * |  | * | 5 |
| Korres et al. (2005a) | * | * |  | * | * |  |  | 4 |
| Li et al. (2016) | * | * | * | * |  |  |  | 4 |
| Lin et al. (2005),  Lin et al. (2007) | * | * |  | * | * |  |  | 4 |
| Liu and Liu (2013) | * | * |  | * | * | * |  | 5 |
| Magnani et al. (2015) | * | * | * | * | * |  |  | 5 |
| Mason and Herrmann (1998) | * | * |  | * | * |  |  | 4 |
| Mehl and Thomson (2002) | * | * |  | * |  | * | * | 5 |
| New York State UNHS project (Prieve et al., 2000) | * | * |  | * | * |  |  | 4 |
| Olusanya et al. (2008), Olusanya (2009) | * |  | * | * | * | * |  | 5 |
| Parab et al. (2018) | * | * |  | * |  |  |  | 3 |
| Park et al. (2020) | * | * |  |  |  | * | * | 4 |
| Pitathawatchai et al. (2019) | * |  | * | * |  | * | * | 5 |
| Prince et al. (2003) | * | * |  | * |  | * |  | 4 |
| Ravi et al. (2021) |  |  |  | * |  |  | * | 2 |
| Razak et al. (2020) | * |  |  | * |  | * | * | 4 |
| Scheepers et al. (2014) | * |  |  | * | * | * |  | 4 |
| Shang et al. (2016) | * | * |  | * | * |  |  | 4 |
| Tanon-Anoh et al. (2010) | * |  |  | * | * | * |  | 4 |
| Thomson and Yoshinaga-Itano (2018) | * | * | * | * |  | * |  | 5 |
| Uilenburg et al. (2009) | * |  |  | * | * |  | * | 4 |
| Vohr et al. (2002) | * | * | * | * | * | * | * | 7 |
| Welzl-Mueller et al. (2001) | * |  |  | * |  |  |  | 2 |
| Wenjin et al. (2018) | * | * | * | * | * |  |  | 5 |
| Wroblewska-Seniuk et al. (2005) | * |  |  | * | * | * |  | 4 |
