# Supplementary material for: Protocol and programme factors associated with referral and loss to follow-up from newborn hearing screening: a systematic review
Source: BMC Pediatr. 2022 Aug 5;22:473. doi: 10.1186/s12887-022-03218-0 (PMC9354382; doi:10.1186/s12887-022-03218-0)
Supplement: Supplementary file 3 — Additional file 3. Overview of all studies included in the systematic review. [file 12887_2022_3218_MOESM3_ESM.docx]

Additional file 3: Overview of all studies included in the systematic review

| **Author/s (year), country** | **Quality** | |  | **Factors investigated** | |  | **Study parameters** | | |  | **Screening protocol parameters** | | | |  | **Findings** | |
| --- | --- | --- | --- | --- | --- | --- | --- | --- | --- | --- | --- | --- | --- | --- | --- | --- | --- |
|  | **RR /6** | **LTFU /7** |  | **Outcomes** | **Determinants** | **Total n**  **(n per group)** | | **Within/Between Subjects** | |  | **Infant groups** | **Method** | **Uni- / Bilat. Refer** | **WB age** |  | **Referral rate** | **LTFU rate** |
| Akinpelu et al. (2019), Canada | 4 |  |  | RR | Passing criteria |  | 255 | | W |  | WB | DPOAE | Uni | Up to discharge |  | 12% using SNR 6 dB in 4/6 freq bands 1.5 to 4 kHz, compared to 4% in all freq bands 4 to 10 kHz |  |
| Arslan et al. (2013), Turkey | 4 |  |  | RR | Age (0-7 days) |  | 2229 | | B |  | All | TEOAE | Uni | 0-7 days |  | Decreased from 34%, 17%, 13%, 8%, 5% to 2% for infants screened on day 0 to 7 |  |
| Augustine et al. (2014), India | 5 | 4 |  | RR+LTFU | WB vs. NICU |  |  | | B |  | WB vs. NICU | aABR | ND | 24-72 hrs |  | 6% for NICU babies compared to 10% for WB babies | 17% for NICU babies compared to 21% for WB |
| Barker et al. (2013), Australia | 5 | 6 |  | RR+LTFU | Stand-alone vs embedded NICU screening |  | 6261 (3346/3015) | | B |  | NICU | aABR | Uni | -- |  | 21.7% for the stand-alone programme compared to 3.9% when NICU screening was incorporated into the universal programme | 0% for infants returning for step 2 at the screening hospital, compared to 14.3% of infants that were scheduled directly for diagnostic assessment. Out of the infants referred from step 2, 4% were LTFU to diagnostic assessment. |
| Benito-Orejas et al. (2008), Spain | 6 | 6 |  | RR+LTFU | TEOAE vs. aABR |  | 5571  (2454/2846) | | B |  | All | TEOAE^R^ vs. aABR^R^ | Uni | 24-48 hrs |  | 3% with aABR compared to 10% with TEOAE | 1% for infants screened with aABR compared to 6% for infants screened with TEOAE |
| Berninger and Westling (2011), Sweden | 5 |  |  | RR | Age (1-7 days) |  | 31092 | | B |  | All | TEOAE | Uni | 1-7 days |  | Decreased from 29%, 14%, 9%, 7% to 6% for infants screened day 1 to 7. |  |
| Bishnoi et al. (2019), India | 1 |  |  | RR | No RF vs. RF |  | 2000 (1594/406) | | B |  | No RF vs. RF | TEOAE | ND | 0-48 hrs |  | 10% for infants with RF compared to 9% for infants without RF |  |
| Botelho et al. (2010), Brazil | 4 | 4 |  | RR+LTFU | WB vs. NICU |  | 6889  (5271/1618) | | B |  | WB vs. NICU | TEOAE | ND | 24-48 hrs |  | 13% for NICU babies compared to 19% for WB | 7% for NICU babies compared to 28% for WB |
| Burdzgla et al. (2007), Georgia | 1 |  |  | RR | Rescreening in step 1 |  | 269 | | W |  | WB | TEOAE | ND | 0-3 days |  | Reduced from 81% to 30% to 8% if rescreening every day from birth to day 2. |  |
| Chalkiadakis et al. (2014), Greece | 3 |  |  | RR | TEOAE vs. aABR |  | 173 | | W |  | NICU | TEOAE vs. aABR | Uni | -- |  | 10% with aABR compared to 13% with TEOAE |  |
| Chan et al. (2015), China | 3 |  |  | RR | Screening device |  | 167 | | W |  | WB | aABR^R^ | Uni | 5-38 hrs |  | 55% with probe-type device compared to 10% with coupler-type device |  |
| Chang et al. (2020), Chung et al. (2019), Chung et al. (2020) | 4 |  |  | RR | WB vs. NICU  TEOAE vs. aABR  Age (0-7+ days) |  | 512982 (57837/2903/448709/ 14793) | | B |  | WB vs. NICU | TEOAE vs. aABR | Uni | 0-7+ days |  | 11% for NICU babies using OAE compared to 3% for WB using OAE. 7% for NICU babies using aABR compared to 1% for WB using aABR.  For WB using aABR, rates were <2% until day 7 when they increased to 3%. For NICU using aABR, rates decreased from 18% on day 1 to 3% on day 7. Rates increased for both groups after day 7. |  |
| Chen et al. (2012), China | 2 | 2 |  | RR+LTFU | no RF vs RF |  | 11568  (10665/ 903) | | B |  | no RF vs RF | TEOAE | Uni | 2-7 days |  | 35% for infants with RF compared to 29% for infants without RF | 49% for infants with RF compared to 24% for infants without RF |
| Ciorba et al. (2008), Ciorba et al. (2007), Italy | 4 |  |  | RR | no RF vs RF |  | 7775  (6759/ 1016) | | B |  | no RF vs RF | TEOAE | Uni | ND |  | 15% for infants with RF compared to 12% for infants without RF |  |
| Clemens and Davis (2001), USA | 6 |  |  | RR | Rescreening in step 1 |  | 3142 | | W |  | WB | aABR | ND | Up to discharge |  | Reduced from 4% to 1% by rescreening before discharge (12-24 hrs after initial screen) |  |
| Connolly et al. (2005), USA | 6 | 6 |  | RR+LTFU | WB vs. NICU |  | 17602  (14416/ 3186) | | B |  | WB vs. NICU | aABR^R^ | Uni | Discharge |  | 7% for NICU infants compared to 3% for WB | 14% for NICU babies compared to 8% for WB |
| Cunningham et al. (2018), USA |  | 5 |  | LTFU | Compliance to guidelines  Screening professional  Step 2 scheduling and fees  Audiologist involved in screening  Step 2 location  Staff training |  | Total N screened ND; Total N referred: 13904 | | B |  | WB | ND | ND | Before discharge |  |  | No significant association was found between LTFU and most hospital factors, including compliance with guidelines, screening professional, step 2 booking procedures, audiologist involvement, step 2 screening location or staff training. |
| Dauman et al. (2009), France | 5 |  |  | RR | WB vs. NICU |  | 113099  (109349/3750) | | B |  | WB vs. NICU | aABR^R^ | ND | Discharge |  | 11% for NICU babies compared to 6% for WB |  |
| De Ceulaer et al. (1999), De Ceulaer et al. (2001) Belgium | 3 | 3 |  | RR+LTFU | WB vs. NICU  Passing criteria |  | 3751 | | B |  | WB vs. NICU  All | TEAOE | Bi | Discharge |  | Approx. 4% annually for NICU babies compared 2 to 2.5% annually for WB.  3% for both strict and lenient passing criteria. | No significant difference in LTFU rate for infants screened with different passing criteria. |
| de Kock et al. (2016), South Africa | 5 | 6 |  | RR+LTFU | DPOAE vs. aABR  Screener experience |  | 7451  (3573/ 3879) | | B |  | All | DPOAE^R^ vs. aABR^R^ | Bi | 6 days (average) |  | 5% with aABR compared to 7% with DPOAE | 13% for infants screened with aABR compared to 10% for infants screened with DPOAE |
| Deniz et al. (2020), Cyrpus | 3 |  |  | RR | Screening device |  | 609 | | B |  | All | aABR | Uni | 1-45 days |  | 20% with GSI AUDIOscreener compared to 24% with MAICO MB11 Beraphone |  |
| Dimitriou et al. (2016), Greece | 3 |  |  | RR | Age (0-5+ days) |  | 2485 | | B |  | WB | TEOAE | Uni | 0 to 5+ days |  | Decreased from 42%, 25%, 10%, to 5% for infants screened day 0 to day 4. 9% for infants screened day 5 or later. |  |
| Doyle et al. (1997), Doyle et al. (1998), USA | 3 |  |  | RR | TEOAE vs. aABR |  | 200 | | W |  | WB | TEOAE vs. aABR | Uni | 0-36+ hrs |  | 18% with aABR compared to 30% with TEOAE |  |
| Erturk et al. (2010), Turkey | 3 | 4 |  | RR+LTFU | TEOAE vs. aABR |  | 500 | | W |  | WB | TEOAE vs. aABR | Uni | 0-9 days |  | 2% with aABR compared to 3% with TEOAE | 11% for infants screened with aABR compared to 21% for infants screened with TEOAE. |
| Fan et al. (2010), Taiwan | 3 |  |  | RR | Hospital size |  | 7139 (3881/1721) | | B |  | WB | aABR^R^ | ND | 0-24 hrs |  | 1.6% for infants screened in a large tertiary hospital, compared to 1.3% for infants screened in smaller maternity hospital and 1.4% for infants screened in community clinics. |  |
| Farhat et al. (2015), Iran | 2 | 2 |  | RR+LTFU | WB vs. NICU |  | 8987  (8724/ 263) | | B |  | WB vs. NICU | TEOAE | ND | Discharge |  | 70% for NICU babies compared to 12% for WB | 3% for NICU babies compared to 21% for WB |
| Finitzo et al. (1998), USA | 6 | 6 |  | RR+LTFU | TEOAE vs. aABR vs. two-tech |  | 52508 (4313/4577 /18121) | | B |  | ALL | TEOAE^R^ vs. aABR^R^ vs. two-tech | Uni | >24 hrs (>48 for c-section) |  | 3% with aABR compared to 10% with TEOAE and 3% with two-tech. | 20% for infants screened with aABR, 29% for infants screened with TEOAE, and 33% for infants screened with two-tech. |
| Fitzgibbons et al. (2021), Australia | 6 | 7 |  | RR+LTFU | No RF vs. RF |  | 613027 (598794/ 14233) | | B |  | No RF vs. RF | aABR^R^ | Uni | Up to discharge (ideally) |  | 7% for infants with risk factors, compared to 1% for infants without risk factors | 14% for infants with risk factors, compared to 11% for infants without risk factors |
| Gabbard et al. (1999), USA | 3 |  |  | RR | TEOAE vs. aABR  Passing criteria |  | 110 | | W |  | WB | TEOAE vs. aABR | Uni | 0-24 hrs |  | 3% with aABR compared to 39% with TEOAE.  For TEOAE, 43% with strict passing criteria compared to 39% for lenient criteria. |  |
| Gallus et al. (2020), Italy | 3 |  |  | RR | Screener experience |  | 12871 | | B |  | WB | TEAOE^R^ | Uni | 1-3 days |  | No consistent trend was found to experience (in months). |  |
| Gilbey et al. (2013), Israel | 4 |  |  | RR | No RF vs. RF |  | 5334 (4958/ 376) | | B |  | No RF vs. RF | TEOAE^R^ | Uni | Discharge |  | 23% for babies with RF compared to 19% for babies without RF | 1% for babies with RF compared to 6% for WB |
| Gina et al. (2021), South Africa | 3 |  |  | RR | TEOAE vs. aABR |  | 2269 | | W |  | WB | TEOAE vs. aABR | ND | 0.6 hrs |  | 7% with aABR compared to 71% with TEOAE |  |
| Govaerts et al. (2001), Belgium | 4 |  |  | RR | WB vs. NICU  Screening device |  | 1995 (1772/223)  1954 (981/973) | | B  B |  | WB vs. NICU  All | TEOAE | Bi | 3-5 days |  | 0.4% for NICU babies compared to 2% for WB.  5% for Echoport device compared to 6% for Echocheck device. |  |
| Grasso et al. (2008), Italy | 4 |  |  | RR | Setting |  | 3303 (1979/1324) | | B |  | WB | TEOAE | Uni | 24+ hrs |  | 25% for infants screened in the general newborn room in one hospital, compared to 10% for infants screened in the mother’s room in a second hospital. |  |
| Hergils (2007), Sweden | 6 |  |  | RR | Hospital size |  | 81648 (69766/11882) | | B |  | All | TEOAE | Uni | 2+ days |  | 16% for infants screened in a large maternity hospital, compared to 20% for infants screened in a small maternity hospital. |  |
| Hrncic et al. (2019), Bosnia & Herzegovina | 4 |  |  | RR | WB vs. NICU  Age (<24 to 48+ hrs) |  | 1217 (884/281)  884 | | B  B |  | WB vs. NICU  WB | TEOAE | Uni | 0-48+ hrs |  | 22% for NICU babies compared to 18% for WB.  Decreased from 29%, 26%, 18%, to 8% for infants screened <24 hrs, 24-36 hrs, 36-48 hrs, or 48+ hrs. |  |
| Hrncic et al. (2021), Bosnia & Herzegovina | 4 | 5 |  | RR+LTFU |  |  | 1217 (930/287) | | B |  | WB vs. NICU | TEOAE | ND | ND |  | 22% for NICU babies compared to 17% for WB | 37% for NICU babies compared to 39% for WB |
| Hsu et al. (2013), Taiwan | 4 |  |  | RR | TEOAE vs. aABR |  | 9110  (5749/3361) | | B |  | WB | TEOAE vs. aABR^R^ | Uni | 1 day (aABR) 3 days (OAE) to discharge |  | 1% with aABR compared to 9% with TEOAE |  |
| Hunter et al. (2016), USA |  | 5 |  | LTFU | Step 2 screening and location |  | Total N screened is ND; Total referrals: 260 | | B |  | WB | ND | ND | Before discharge |  |  | LTFU was significantly lower when step 2 screening was implemented at accessible Women, Infants and Children’s locations, compared to control groups in which infants who failed step 1 were referred for diagnostic assessment |
| Identification of neonatal hearing impairment project (Norton et al., 2000a, Norton et al., 2000b, Norton et al., 2000c, Sininger et al., 2000), USA | 5 |  |  | RR | WB vs. NICU  TEOAE vs. aABR vs. DPOAE  Passing criteria |  | (2348/4478) | | B  W |  | WB vs. NICU  WB & NICU | TEOAE vs. aABR vs. DPOAE | Uni | Varies (approx. 1-10 days) |  | For WB: 13% with aABR compared to 15% with TEOAE and 16% with DPOAE. For NICU: 14% with aABR compared to 15% with TEOAE and 18% with DPOAE.  Specificity from 0 to 100% is plotted a function of SNR passing criteria for TEOAE (-40 to 30 SNR) and Fsp for aABR (0.1 to 40). |  |
| Isaacson (2000), USA | 4 |  |  | RR | no RF vs RF |  | 2031 (1618/413) | | B |  | no RF vs RF | TEOAE^R^ | Uni | 16 hrs-discharge |  | 14% for babies with RF compared to 7% for babies without RF |  |
| Jacob et al. (2021), India | 2 | 2 |  | RR+LTFU | no RF vs. RF |  | 773 (472/301) | | B |  | No RF vs. RF | TEOAE | ND | Up to discharge (ideally) |  | 19% for babies with RF compared to 11% for babies without RF | 54% for babies with RF compared to 54% for babies without RF |
| Januario et al. (2015), Brazil | 4 | 4 |  | RR+LTFU | no RF vs RF |  | 6987 (6082/905) | | B |  | no RF vs RF | TEOAE | Uni | 23 days |  | 15% for babies with RF compared to 7% for babies without RF | 26% for babies with RF compared to 30% for babies without RF |
| Kanji et al. (2018), South Africa | 2 |  |  | RR | Infant age, inpatient vs outpatient |  | 367  (99/268) | | B |  | WB | DPOAE | Uni | <6 hrs vs. 3 days |  | 1% for infants screened 3 days after birth in outpatient setting compared to 84% for infants screened in the maternity ward from 0-6 hours after birth. |  |
| Kelly et al. (2021), USA | 4 |  |  | RR | Age (0-48 hrs) |  | 31984 | | B |  | WB | aABR | Uni | 0-48 hrs |  | 22% at 0-8 hrs decreased to 11% at 39-48 hrs. |  |
| Kennedy et al. (1991), U.K. | 3 |  |  | RR | TEOAE vs. aABR |  | 370 | | W |  | All | TEOAE vs. aABR | Uni | ND |  | 3% with TEOAE and 3% with aABR |  |
| Kennedy et al. (2000), U.K. | 2 |  |  | RR | TEOAE vs. aABR |  | 37452 (25199/4478) | | B |  | WB | TEOAE^R^ vs. two-tech | Bi | ND |  | 1% with two-technology screening compared to 2% with TEOAE-only |  |
| Kishino et al. (2021), Japan | 3 |  |  | RR | Screening device |  | 1744 (267/1477) | | B |  | no RF | aABR | Uni | 0-7 days |  | 4% with MAICO MB11 Beraphone compared to 1% with Natus ALGO2e color. |  |
| Kolski et al. (2007), France | 3 | 3 |  | RR+LTFU | Infant age, inpatient vs outpatient |  | 4726 (3065/1661) | | B |  | WB | TEOAE | Bi | Discharge vs. 2 mo |  | 3% for infants screened at an outpatient clinic 2 months after birth, compared to 1% for infants screened in the maternity hospital at discharge. | 35% of infants screened at an outpatient clinic 2 months after birth, compared to 6% screened at the maternity hospital at discharge. Step 2 screening performed several weeks later at original screening location. |
| Konukseven et al. (2010), Turkey | 5 |  |  | RR | TEOAE vs. aABR |  | 1917 | | W |  | WB | TEOAE vs. aABR | Uni | 0-48 hrs |  | 2% with aABR compared to 11% with TEOAE |  |
| Korres et al. (2003a), Greece | 4 | 5 |  | RR+LTFU | Passing criteria |  | 5295 | | B |  | WB | TEOAE^R^ | Uni | 2 days |  | 3.2% for strict passing criteria and 4% for lenient passing criteria. | No significant difference for infants screened with different passing criteria. |
| Korres et al. (2003b), Greece | 2 |  |  | RR | Age (1-4 days) |  | 2121 (4 groups) | | B |  | no RF | TEOAE | Uni | 1-4 days |  | Reduced from 15%, 9%, 6% to 3% for infants screened on day 1 to day 4. |  |
| Korres et al. (2005a), Greece | 4 | 4 |  | RR+LTFU | Infant status  Rescreening in step 1  Passing criteria |  | 22195 | | B |  | WB | TEOAE^R^ | Uni | 2 days |  | Reduced from 9.8% to 5.9% when screening was postponed due to infant being noisy.  2.4% for strict passing criteria and 2.5% for lenient criteria.  Reduced from 5.2% to 3.6% after immediate rescreening. | No significant differences in LTFU for any factors investigated. |
| Korres et al. (2005b), Greece | 3 |  |  | RR | WB vs. NICU |  | 25288 (23574/1715) | | B |  | WB vs. NICU | TEOAE^R^ | Uni | Up to discharge |  | 7% for NICU babies compared to 2% for WB |  |
| Korres et al. (2006), Greece | 3 |  |  | RR | TEOAE vs. aABR |  | 100 | | W |  | WB | TEOAE vs. aABR | Uni | ND |  | 23% with aABR compared to 21% with TEOAE |  |
| Labaeka et al. (2018), Nigeria | 3 |  |  | RR | Age (admission vs. discharge) |  | 201 | | W |  | NICU | aABR | Uni | -- |  | 41% at NICU admission reduced to 16% at NICU discharge. |  |
| Li et al. (2016), Taiwan | 4 | 4 |  | RR+LTFU | WB vs. NICU |  | 15586 (13645/1941) | | B |  | WB vs. NICU | aABR^R^ | Uni | 48 hrs to discharge |  | 3% for NICU babies compared to 1% for WB | 11% for NICU babies compared to 23% for WB |
| Lin et al. (2005), Lin et al. (2007), Taiwan | 4 | 4 |  | RR+LTFU | TEOAE vs. aABR vs. two-tech |  | 25588 (18260/3788/ 3540) | | B |  | WB | TEOAE^R^ vs. aABR^R^ vs. two-tech | Uni | 48 hrs to discharge |  | 0.8% with aABR compared to 6% with TEOAE and 2% with two-tech | 10% for infants screened with aABR compared to 19% for infants screened with OAE and 30% for infants screened with two-tech. |
| Liu and Liu (2013), China | 5 | 5 |  | RR+LTFU | WB vs. NICU |  | 11894 (8321/3573) | | B |  | WB vs. NICU | TEOAE | Uni | 3-5 days |  | 22% for NICU babies compared to 15% for WB | 38% for NICU babies compared to 68% for WB |
| Magnani et al. (2015), Italy |  | 5 |  | LTFU | no RF vs RF |  | 11592 (10359/1233) | | B |  | no RF vs RF | Varies | Uni | 24-48 hrs |  |  | 14% for infants with RF, compared to 8% for infants without RF. |
| Martines et al. (2007), Martines et al. (2012), Italy | 4 |  |  | RR | no RF vs RF |  | 3379 (3048/331) | | B |  | no RF vs RF | TOEAE^R^ | Uni | 20 days |  | 7% for infants with RF compared to 2% for infants without RF |  |
| Mason and Herrmann (1998), USA | 4 | 4 |  | RR+LTFU | WB vs. NICU |  | 10372 (8971/1401) | | B |  | WB vs. NICU | aABR | Bi | 24-36 hrs |  | 5% for NICU babies compared to 4% for WB | 6% for NICU babies compared to 10% for WB |
| McPherson et al. (2006), China | 3 |  |  | RR | TEOAE vs. TBOAE |  | 298 | | W |  | WB | TEOAE vs. TBOAE | Uni | 1-7 days |  | No significant difference TE vs. TBOAE |  |
| Mehl and Thomson (2002), USA | 4 | 5 |  | RR+LTFU | Hospital size |  | 50414  (4 groups) | | B |  | All | Varies (most aABR) | ND | Up to discharge |  | 6% for hospitals with 100-400 births/year, 1% for 400-1000 births/year, 2% for 1000-2000 births/year and 2% for 2000-6000 births/year | 37% for hospitals with 100-400 births/year, 31% for 400-1000 births/year, 32% for 1000-2000 births/year and 19% for 2000-6000 births/year |
| Meyer et al. (1999), Germany | 3 |  |  | RR | TEOAE vs. aABR |  | 464 | | W |  | RF | TEOAE vs. aABR | Uni | 2-7 days |  | 5% with aABR compared to 30% with TEOAE |  |
| Murray et al. (2004), USA | 3 |  |  | RR | Screening device |  | 193 | | W |  | WB | aABR^R^ | Uni | Varies (4- 24+ hrs) |  | 6% with Natus ALGO3 compared to 11% with ALGO2e. After rescreening before discharge, differences were negligible. |  |
| New York State UNHS project (Gravel et al., 2000, Spivak et al., 2000, Prieve et al., 2000), USA | 4 | 4 |  | RR+LTFU | WB vs. NICU  TEOAE vs. two-tech |  | (60356/11566) | | B |  | WB vs. NICU  WB & NICU | TEOAE^R^ vs. two-tech | Uni | Up to discharge |  | 6% for NICU babies compared to 4% for WB.  For WB: 2% with two-tech compared to 7% with TEOAE-only. For NICU: 5% with two-tech compared to 8% with TEOAE-only | 24% for NICU babies compared to 28% for WB |
| Nishad et al. (2020), India | 2 |  |  | RR | No RF vs. RF |  | 1000 (693/307) | | B |  | No RF vs. RF | OAE | Uni | 3 days |  | 16% for babies with RF compared to 10% for babies without RF |  |
| Olusanya et al. (2008), Olusanya (2009), Nigeria | 5 | 5 |  | RR+LTFU | WB vs. NICU |  | 1330 (1150/180) | | B |  | WB vs. NICU | TEOAE^R^ | Uni | >24 hrs |  | 32% for both NICU and WB | 63% for NICU babies compared to 89% for WB |
| Olusanya (2010), Nigeria | 5 |  |  | RR | Setting (ambient noise) |  | 11897 (2389/783/ 1619) | | B |  | All | TEOAE | Uni | 1 week to 3 months |  | 11%, 18%, 7% and 9% across 4 outpatient screening locations with ambient noise of 63.1, 63.4, 65.3, and 67.9 dBA Leq. |  |
| Olusanya et al. (2009), Nigeria | 4 |  |  | RR | Infant age, inpatient vs outpatient |  | 3333 (1330/2003) | | B |  | All | TEOAE | Uni | 2.6 days (avg) vs. 17 days (avg) |  | 14% for infants screened in an outpatient setting at 17 days, compared to 32% for infants screened in the maternity hospital at 2 days. |  |
| Ong et al. (2020), Philippines | 5 |  |  | RR | TEOAE vs. aABR |  | 247 | | W |  | All | TEOAE vs. aABR | Uni | ND |  | 19% with aABR compared to 11% with TEOAE |  |
| Parab et al. (2018), India | 3 | 3 |  | RR+LTFU | no RF vs RF |  | 8192 (6509/1683) | | B |  | no RF vs RF | TEOAE | ND | 24-72 hrs |  | 15% for infants with RF compared to 24% for infants without RF | 8% for infants with RF compared to 2% for infants without RF |
| Park et al. (2020) | 3 | 4 |  | RR+LTFU | Programme organsiation |  | 239632 (37486/ 202146) | | B |  | All | Various | Uni | ND |  | 2.5% for the area-based programme compared to 1.4% for the nationally organised coupon-mediated programme | 87.6% for the area-based programme compared to 66.5% for the nationally organized coupon-mediated programme |
| Pastorino et al. (2005), Italy | 3 |  |  | RR | Rescreening in step 1 |  | 19290 | | W |  | WB | TEOAE | ND | 36-48 hrs |  | 2.4% from initial screening reduced to 1.2% after rescreening before discharge. |  |
| Pisacane et al. (2013), Italy | 5 |  |  | RR | WB vs. NICU |  | 146026 (141910/4116) | | B |  | WB vs. NICU | TEOAE | Bi | 48-72 hrs |  | 8% for NICU babies compared to 2% for WB |  |
| Pitathawatchai et al. (2019), Thailand | 4 | 5 |  | RR+LTFU | No RF vs. RF |  | 5922 (5612/310) | | B |  | No RF vs. RF | OAE | Bi | Day before discharge |  | 19% for infants with RF compared to 3% for infants without RF | 21% for infants with RF compared to 27% for infants without RF |
| Prince et al. (2003), USA |  | 4 |  | LTFU | Hospital size |  | 10328; Total referrals: 1013 | | B |  | All | ND | ND | ND |  |  | Infants born in smaller community hospitals were less likely to complete follow-up compared to infants born in large maternity centers. |
| Razak et al. (2020), USA |  | 4 |  | LTFU | WB vs. NICU |  | Total ND;  197 referrals (153/44) | | B |  | WB vs. NICU | Various^R^ | Uni | Up to discharge |  |  | 30% for NICU babies compared to 14% for WB. NICU stay > 5 days is a significant risk factor for LTFU in multivariate regression. |
| Ravi et al. (2021), India | 1 | 2 |  | RR+LTFU | WB vs. NICU |  | 1366 (683/683) | | B |  | WB vs NICU | TEOAE | ND | 24-48 hrs / condition stable |  | 18% for NICU babies compared to 4% for WB | 70% for NICU babies compared to 80% for WB |
| Scheepers et al. (2014), South Africa | 4 | 4 |  | RR+LTFU | Hospital size  Age (3.1 vs. 1.8 days) |  | 3089 (954/2135) | | B |  | WB | DPOAE | Uni | 1.8 vs 3.1 days (avg) |  | 21% for infants screened in the large hospital at an average age of 1.8 days, compared to 12% for infants screened in the smaller hospital at an average age of 3.1 days | 64% from the hospital with a higher birth rate, compared to 56% from the smaller hospital. The increased stress on screeners to screen all infants before discharge likely contributed to the higher LTFU. |
| Sergi et al. (2001), Italy | 3 |  |  | RR | no RF vs RF |  | 6517 (5650/867) | | B |  | no RF vs RF | TEOAE | Uni | 36-48 hrs |  | 12% for infants with RF compared to 4% for infants without RF |  |
| Shang et al. (2016), China | 4 | 4 |  | RR+LTFU | TEOAE vs. two-tech |  | 1062 | | W |  | WB | TEOAE vs. two-tech | Uni | 48-72 hrs |  | 4% with two-tech compared to 11% with TEOAE-only | 5% for infants screened with two-tech compared to 4% for infants screened with TEOAE. |
| Shoup et al. (2005), USA | 6 |  |  | RR | Rescreening in step 1 |  | 31681 | | B |  | All | aABR | Uni | 4 hrs to discharge |  | 0.8% when screening at 4 hrs and rescreening before discharge, compared to 0.7% when a 3^rd^ screen was added between initial and pre-discharge screening |  |
| Stewart et al. (2000), USA | 3 |  |  | RR | Screening professional |  | 11711 | | B |  | All | aABR^R^ | Uni | 20-30 hrs (average) |  | No association between referral rate and screening professional across sites |  |
| Tabrizi et al. (2017), Iran | 2 |  |  | RR | Age (0-9 days) |  | 1396 | | B |  | No RF | TEOAE | Uni | 0-9 days |  | Decreases from 11% to 8% for infants screened from 0-3 days and from 3-6 days. |  |
| Tanon-Anoh et al. (2010), Cote d’Ivoire | 4 | 4 |  | RR+LTFU | WB vs NICU |  | 1306 (1156/150) | | B |  | WB vs. NICU | TEOAE | ND | 3-28 days |  | 64% for NICU infants compared to 16% for WB | 13% for NICU babies compared to 43% for WB |
| Tatli et al. (2007), Turkey | 4 |  |  | RR | WB vs NICU |  | 711  (475/236) | | B |  | WB vs. NICU | TEOAE^R^ | Uni | Discharge |  | 9% for NICU infants compared to 2% for WB |  |
| Thomson and Yoshinaga-Itano (2018), USA |  | 5 |  | LTFU | TEOAE vs. aABR  Screening professional  Referral rate  Step 2 scheduling  Step 1 setting  Hospital size  Step 2 location  Audiologist involved in screening |  | 67261 | | B |  | All | Varies |  |  |  |  | Screening method: lost significance once audiologist involvement was incorporated.  Screening professional: only significant in hospitals where an audiologist is not involved in the screening programme. LTFU rates were lowest in places where technicians are the screeners, when compared to nurses and volunteers. Low LTFU rates are achieved using volunteer and nurse screeners with audiologist supervision.  Scheduling step 2: Higher LTFU when parents were responsible for booking the step 2 screening, compared to if the booking was done prior to hospital discharge. This variable was directly related to whether an audiologist was involved in the NHS programme  Step 1 setting: Infants born in hospitals with a NICU were 38% more likely to attend step 2 screening. This trend was directly correlated to the presence of audiology involvement Hospital size: Hospitals with birth rates >3000 had the lowest LTFU. Hospitals with birth rates from 2000-3000 had the highest LTFU. These also had more volunteer screeners, and parents were mostly responsible for scheduling the step 2 appointment. Trends lost significance once audiologist involvement was incorporated.  Referral rate: Involvement of an audiologist in the screening programme removed any associative trend.  Step 2 location: Hospitals that referred families to an external audiology clinic for step 2 screening had a higher LTFU rate compared to if families returned to the initial screening location. LTFU was even lower if the screening hospital had an audiology department  Audiologist involvement: Programmes with an audiologist involved had lower LTFU. |
| Tzanakakis et al. (2016), Greece | 5 |  |  | RR | TEOAE vs. DPOAE |  | 3480 | | W |  | All | TEOAE vs. DPOAE | Uni | Varies |  | 26% with DPOAE compared to 9% with TEOAE |  |
| Uilenburg et al. (2009), Netherlands | 3 | 4 |  | RR+LTFU | Infant age, inpatient vs outpatient  Step 2 location |  | 3137 (1482/770/885) | | B |  | WB | TEOAE^R^ | Uni | 4-7 days vs. 3-4 weeks |  | 6% for infants screened at home at 4-7 days, compared to 8% when screened at home at 3-4 weeks and 8% when screened at an outpatient clinic at 3-4 weeks. | No infants were LTFU after being screened at home at 4-7 days, compared to 8% when screened at home at 3-4 weeks and 10% when screened at an outpatient clinic at 3-4 weeks. Step 2 screening occurred after 1 week in the same location as step 1. |
| Vernier et al. (2021), Brazil | 3 |  |  | RR | Age (24 vs. 36 hrs)  Rescreening in step 1 |  | 462 | | W |  | no RF | TEOAE | Uni | 24 vs. 36 hrs |  | 29% at 24 hrs reduced to 15% when rescreened at 36 hrs. An immediate rescreening at 36 hrs after pinna manipulation and reinsertion reduced rate from 15% to 9%. |  |
| Vignesh et al. (2015), India | 3 |  |  | RR | No RF vs. RF |  | 1405 (983/422) | | B |  | no RF vs. RF | Two-tech | Uni | 2-28 days |  | 5% for infants with RF compared to 1% for infants without RF |  |
| Vohr et al. (1993), USA | 1 |  |  | RR | Age (1-4+ days)  Infant status |  | 3939 | | B |  | WB | TEOAE | ND | 1-4+ days |  | Decreased from 32%, 21%, 19%, to 19% for infants screened from day 1 to day 4+.  Decreased from 42% when performed during crying, to 21% when screening performed while quiet. |  |
| Vohr et al. (2002), USA |  | 7 |  | LTFU | WB vs. NICU |  | 39153 (35254/3680) | | B |  | WB vs. NICU | Two-tech^R^ | Uni | Up to discharge |  |  | NICU infants are almost 6 times more likely to be LTFU (95% CI 2.55-9.98) |
| Welzl-Mueller et al. (2001), Austria | 2 | 2 |  | RR+LTFU | WB vs. NICU |  | 6662 (5857/805) | | B |  | WB vs. NICU | TEOAE^R^ | ND | Up to discharge |  | 4% for NICU infants compared to 1% for WB | 45% for NICU infants compared to 28% for WB |
| Wenjin et al. (2018), China |  | 5 |  | LTFU | WB vs. NICU |  | 19098 (12134/6964) | | B |  | WB vs. NICU | Varies | Uni | Discharge |  |  | 41% for NICU babies compared to 28% for WB |
| Wessex Universal Neonatal Hearing Screening Trial Group (1998), U.K. | 4 |  |  | RR | Age (1-4+ days) |  | 21279 | | B |  | All | TEOAE | Bi | 1-4+ days |  | Decreases from 5%, 2% to 1% for infants screened on day 1 to 4; 3% for infants screened after day 4 |  |
| Wroblewska-Seniuk et al. (2005), Wroblewska-Seniuk et al. (2017), Poland | 4 | 4 |  | RR+LTFU | no RF vs. RF |  | 6827 (6055/772) | | B |  | no RF vs. RF | TEOAE^R^ | Uni | Discharge |  | 7% for infants with RF compared to 1% for infants without RF | 83% for infants with RF compared to 64% for infants without RF. |
| Yilmazer et al. (2016), Turkey | 4 |  |  | RR | no RF vs. RF |  | 5985 (4111/1874) | | B |  | no RF vs. RF | TEOAE | Uni | 1-2 days |  | 28% for infants with RF compared to 32% for infants without RF |  |
| Yoshikawa et al. (2004), Japan | 2 |  |  | RR | WB vs. NICU |  | 226 (124/102) | | B |  | WB vs. NICU | aABR | Uni | ND |  | 8% for NICU babies compared to 1% for WB |  |

RR: referral rate; LTFU; lost to follow-up; WB: well baby; NICU: neonatal intensive care unit; RF: risk factors for permanent hearing impairment; ND: not described; B: between-subject comparison; W: within-subject comparison; TEOAE: transient-evoked otoacoustic emissions: aABR: automated auditory brainstem response; Two-tech: two-technologies are used in sequence within one screening step (i.e., OAE and then aABR for infants who do not pass OAE); ^R^ indicates that the screening test is repeated immediately or some hours later in the same screening step / before discharge from the maternity hospital. With a unilateral referral a screening fail in one ear only is sufficient to warrant referral to step 2 / diagnostic assessment; with bilateral referral, a failed screen in both ears is required for referral.
