# Supplementary material for: Protocol and programme factors associated with referral and loss to follow-up from newborn hearing screening: a systematic review
Source: BMC Pediatr. 2022 Aug 5;22:473. doi: 10.1186/s12887-022-03218-0 (PMC9354382; doi:10.1186/s12887-022-03218-0)
Supplement: Supplementary file 4 — Additional file 4. Studies comparing referral rates across screening methods. [file 12887_2022_3218_MOESM4_ESM.docx]

Additional file 4: Studies comparing referral rates across screening methods

| Author/s (year) | Quality rating (/6) | Screening parameters | | | | | | | | Results | | | | |
| --- | --- | --- | --- | --- | --- | --- | --- | --- | --- | --- | --- | --- | --- | --- |
|  |  | Infant group | Age at screening (inpatient vs outpatient) | Passing criteria | | Sample size (n) | | | | Referral rate | | | | Statistics |
|  |  |  |  | OAE | aABR | TEOAE | aABR | DPOAE | Two-tech | TEOAE | aABR | DPOAE | Two-tech |  |
| Benito-Orejas et al. (2008) | 6 | All | >24-48 hrs (inpatient) | WWR+SNR | 35 dB | 2454 | 2846 |  |  | 10% | 3% |  |  | χ^2^=121.3, df=1, p<0.001 |
| Chalkiadakis et al. (2014) | 3 | NICU | Inpatient | Automatic | -- | 173* | 173* |  |  | 13% | 10% |  |  |  |
| Chang et al. (2020), Chung et al. (2019), Chung et al. (2020) | 4 | WB  NICU | Various  Various | Various  Various | ND  ND | 57837  2903 | 448709  14793 |  |  | 3%  11% | 1%  7% |  |  | χ^2^=1387, df=1, p<0.001  χ^2^=71.0, df=1, p<0.001 |
| Doyle et al. (1997) | 3 | WB | 5-48 hrs (inpatient) | WWR or SNR | 35 dB | 200* | 200* |  |  | 30% | 18% |  |  |  |
| Erturk et al. (2010) | 3 | WB | 2 to 220 hrs (inpatient) | Automatic | 35 dB | 500* | 500* |  |  | 3% | 2% |  |  |  |
| Finitzo et al. (1998) | 6 | All | >24 hrs (inpatient) | WWR+SNR | 35 dB | 4313 | 4577 |  | 18121 | 10% | 3% |  | 3% | χ^2^=501.6, df=2, p<0.001 |
| Gabbard et al. (1999) | 3 | WB | <24 hrs (inpatient) | WWR+SNR | 35 dB | 110* | 110* |  |  | 39% | 3% |  |  |  |
| Gina et al. (2021) | 3 | WB | 0-6 hrs (inpatient) | Automatic | 35 dB | 2269* | 2269* |  |  | 71% | 7% |  |  |  |
| Hsu et al. (2013) | 4 | WB | > 1 (aABR) or 3 days (TEOAE) (inpatient) | Not defined | 35 dB | 5749 | 3361 |  |  | 9% | 1% |  |  | χ^2^=232.6, df=1, p<0.001 |
| Identification of Neonatal HI studies (2000) | 5 | WB  NICU | Various (inpatient)  Various (inpatient) | SNR  SNR | 30 dB  30 dB | 2348*  4478* | 2348*  4478* | 2348*  4478* |  | 15%  15% | 13%  14% | 16%  19% |  |  |
| Kennedy et al. (1991) | 3 | All | ND | POEMS | 35 dB | 370* | 370* |  |  | 3% | 2.7% |  |  |  |
| Konukseven et al. (2010) | 5 | WB | <48 hrs (inpatient) | SNR | 40 dB | 1917* | 1917* |  |  | 11% | 2% |  |  |  |
| Korres et al. (2006) | 3 | WB | ND | SNR | -- | 100* | 100* |  |  | 21% | 23% |  |  |  |
| Lin et al. (2005, 2007) | 4 | WB | >48 hrs (inpatient) | SNR | 35 dB | 18260 | 3788 |  | 3540 | 6% | 0.8% |  | 2% | χ^2^=249.0, df=2, p<0.001 |
| Meyer et al. (1999) | 3 | RF | >2-7 days (inpatient) | WWR | 35 dB | 464* | 464* |  |  | 30% | 5% |  |  |  |
| Ong et al., (2020) | 5 | All | ND | Automatic | 35 dB | 247* | 247* |  |  | 11% | 19% |  |  |  |
| De Kock et al. (2016) | 5 | All | avg 6.1 days (outpatient) |  | 35 dB |  | 3879 | 3573 |  |  | 5% | 7% |  | χ^2^=19.5, df=1, p<0.001 |
| Tzanakakis et al. (2016) | 5 | All | ND (inpatient or outpatient) | Automatic |  | 3480* |  | 3480* |  | 9% |  | 26% |  | χ^2^=341.1, p<0.001 |
| McPherson et al. (2006) | 3 | WB | 1 to 7 days (inpatient) | WWR+SNR |  | 298* |  | 298*† |  | ND |  | ND |  | p=0.824 |
| Kennedy et al. (2000) | 2 | WB | ND | ND |  | 25199 |  |  | 12253 | 2% |  |  | 1% | χ^2^=121.5, df=1, p<0.001 |
| Shang et al. (2016) | 4 | WB | >48 hrs (inpatient) | Automatic |  | 1062* |  |  | 1062* | 11% | 4% |  |  |  |
| NY State UNHS project (2000) | 4 | WB  NICU | Various (inpatient)  Various (inpatient) | SNR  SNR |  | ND  ND |  |  | ND  ND | 7%  8% |  |  | 2%  5% | χ^2^=489, df=1, p<0.001  χ^2^=26.8, df=1, p<0.001 |

*same infants were tested with all methods; †tone-burst OAE. Referral rates were calculated for the studies in which the number of passes or referrals were reported and the number of infants screened. Chi-square analyses (χ^2^) were performed to determine a significant difference between groups using SPSS v. 26, if statistics were not provided in the study. Analyses for paired data (i.e., within-subject designs) were not performed since individual-level data were not available.

Two-tech: a two-technology protocol where OAE is performed first followed by aABR on infants that do not pass the OAE test; NICU: infants admitted to the neonatal intensive care unit, WB: well babies, RF: infants with risk factors, WWR: whole-waveform repeatability, SNR: signal to noise ratio, ND: not described.
