# Supplementary material for: Protocol and programme factors associated with referral and loss to follow-up from newborn hearing screening: a systematic review
Source: BMC Pediatr. 2022 Aug 5;22:473. doi: 10.1186/s12887-022-03218-0 (PMC9354382; doi:10.1186/s12887-022-03218-0)
Supplement: Supplementary file 5 — Additional file 5. Summary of findings for programme determinants, divided into protocol-, individual- and organizational-levels. [file 12887_2022_3218_MOESM5_ESM.docx]

Additional file 5: Summary of findings for programme determinants, divided into protocol-, individual- and organizational-levels

|  | **Referral rate** | | **Lost to follow-up (LTFU)** |
| --- | --- | --- | --- |
|  | **OAE** | **aABR** |  |
| *PROTOCOL-LEVEL FACTORS* | | | |
| **Screening method** |  |  | Out of the seven studies investigating LTFU across infants screened with different protocols, only two showed significant differences, though trends were not consistent between these studies [1, 2]. Thomson and Yoshinaga-Itano [3] showed that the effect of screening method on LTFU was significant when unadjusted but lost significance once audiologist involvement was incorporated into the analysis. |
| **Screening device** | No differences were observed between screening devices (Otodynamics Echocheck vs. Echoport) [4]. | No differences were observed between screening devices using the same method (Natus ALGO 2e vs. 3) [5]. Differences were observed between insert-style (Natus EchoScreen) and coupler-stype (Natus ALGO3i) devices [6]. Two devices with coupler-style attachments had referral rates of 4% (Maico MB11) and 1% (Natus ALGO2e color), though differences were not significant [7]. Negligible differences were also noted between two devices using the click versus chirp stimuli [8]. | No included studies measured LTFU between screening devices. |
| **Passing criteria** | No differences were observed between strict (3 dB SNR) and lenient (6 dB SNR) passing criteria [9-12]. No differences also observed between two screening modes in the Otodynamics ILO 88 software [13]. Using high-frequencies only for DPOAEs (4-10 kHz) reduced referral rates from 12% to 4% compared to default screening frequency bands.  Specificity to increasing SNR criteria was modelled in the *Identification of Neonatal HI project* [14]. | Differences were observed between passing criteria at 30 vs. 50 dB nHL Out of the 1243 infants that failed screening at 30 dB nHL in one or both ears, 60% would have passed if screening was instead performed at 50 dB nHL [15].  Specificity to increasing Fsp was modelled in the Identific*ation of Neonatal HI project* [14]. | Three studies reported LTFU after referral using different OAE criteria; no study showed any significant differences in LTFU rates [10-12]. |
| **Rescreening in step 1** | Two studies showed a decrease in referral rate when immediate rescreen was performed after screening fail. In only study referral rate reduced from 5.2 to 3.6% [11]. In a second study at 36 hours, referral rate decreased from 15% to 9% after pinna manipulation and reinsertion of the probe. A decrease was also observed when a rescreen was added just before discharge [16, 17]. | A decrease was observed if a rescreen is added before discharge [18]. A marginal decrease in referral rate at discharge was observed if a 3^rd^ screen was added 24 hours after initial screen (where a pre-discharge rescreen already exists) [19]. | Neither study that reported LTFU after step 1 showed a significant difference in LTFU rate when rescreening was performed prior to discharge [11, 19]. |
| *INDIVIDUAL-LEVEL FACTORS* | | | |
| **Infant status** | A decrease was observed if screening is only performed when infants are quiet [11, 13].  A protocol adjustment to postponed screening if the infant status was not ideal for testing (i.e., if the infant was upset or making noise) reduced referral rates from 9.8% to 5.9% [11]. | No included studies investigated infant status with aABR. | LTFU rates were not affected by infant status [11]. |
| **Infant age since birth as inpatient (days)** | A consistent decrease was observed from day 0 to day 3 [13, 20-28]. Findings on the relative referral rate varied across studies after day 3. Scheepers et al. who compared WB screening between two hospitals, one that screened at an average of 1.8 days of age (referral rate of 21%) and another that screened at 3.1 days of age (referral rate of 12%) [27]. | In the population-based study by Chung et al., 90% of WBs are screened with aABR [29]. Small differences were observed from day 0 to day 7 (0.4 to 1.5%). From day 7 to day 60, rates increase from 2.9 to 4.7%. Another single-center study found a reduction from <8 hours (22%) to 39-48 hours after birth (11%) [30].  For NICU infants, lower aABR referral rates were found at discharge (16%) versus NICU admission (41%) [31]. Chung et al. showed a reduction from day 1 to day 7 of 17.9% to 2.9% and subsequent increase after day 7. | Scheepers et al. reported higher LTFU for one busier hospital where infants were screened at a younger mean age; this could be related to stress on the screeners to screen more children (described later) [27]. |
| **Infant age, inpatient versus outpatient** | For two studies, lower referral rates were observed from an outpatient setting compared to inpatient when screening was performed within a few weeks of age [32, 33]. Conversely, one study found slightly higher referral rates when screening was performed at 2 months of age compared to discharge [34]. No significant differences in referral rates were observed for infants aged 4-7 days versus 2-4 weeks when performed in the same location [35]. | No included studies investigated referral rates from aABR in an inpatient versus outpatient setting. | LTFU rates after screening referral was 6% from the maternity ward compared to 35% from an outpatient clinic setting [34]. A slightly higher LTFU rate was observed when screening was performed in a well-baby clinic compared to at home [35]. |
| **Screening professional, experience and training** | de Kock et al. showed that across two screeners, one with more experience had lower referral rate compared to the other with less experience [36].  In another study, no systematic learning curve was observed from the 1st to 12^th^ month of screening for four groups of three newly trained screeners.  No included study investigated referral rates across screening professionals. | In contrast to OAE findings, de Kock et al. showed that the screener with more experience did not have lower referral rates compared to the less experienced screener with aABR [36].  No differences were observed across screening professionals [37]. | Thomson and Yoshinaga-Itano found an association between screening professional and LTFU rate only in hospitals where an audiologist is not involved in the screening programme [3]. LTFU rates were lowest in places where technicians are the screeners, when compared to nurses and volunteers. Low LTFU rates are achieved using volunteer and nurse screeners with audiologist supervision.  In Cunningham et al, there was audiologist involvement in all but 7 hospitals. This study found no association between screening professionals and LTFU rate [38]. They also found no association with regards to whether screeners were trained on counselling parents. This supports the necessity for audiologist involvement in NHS. |
| **Audiologist involvement** |  |  | This was the most influential factor in the population-based multivariate analysis performed by Thomson and Yoshinaga-Itano [22]. Hospital programmes that had an audiologist involved had lower LTFU, and inclusion of this factor in the model decreased the strength and significance of association for other factors, such as technology and screening professional.  This factor did not have a significant association to LTFU in the study by Cunningham et al. [38]. However, as described, the bulk of hospitals in this later study had an audiologist involved in NHS. The other difference between studies was the exclusion of NICU infants from Cunningham et al. |
| *ORGANISATIONAL-LEVEL FACTORS* | | | |
| **Setting and hospital size** | Ambient noise levels (63.1 to 67.9 dB Leq) were not related to referral rates [39]. Referral rates were lower when infants were screened in the mother’s room compared to a newborn nursery [39].  Hergils reported slightly but significantly lower referral rates for one larger hospital compared to a smaller hospital [40]. Smaller than expected differences could be due to the support and training provided to smaller facilities by bigger and more experienced centres.  Scheepers et al. reported a higher referral rate for a hospital with higher birth rate compared to a hospital with lower birth rate [27]. Infants screened in the busier hospital were younger, on average. | Mehl and Thomson reported an overall referral rate of 6% for 16 hospitals with annual birth rates of 400 to 1000 compared to 1-2% for hospitals with annual birth rates >1000 [41]. This was likely attributed to the increased experience and practice with screening methods among professionals testing more babies in larger hospitals.  In contrast, Fan et al. showed no differences in referral rates between type of facility (a tertiary care hospital, smaller maternity hospital, and birth clinics) [42]. Referral rates for all three settings were already <2%. | Three studies showed lower LTFU rate among bigger hospitals [3, 41, 43].  Thomson and Yoshinaga-Itano also found that hospitals with annual birth rates from 2000-3000 had the highest LTFU rates. These hospitals also had more volunteer screeners, and parents were mostly responsible for scheduling the step 2 appointment. Hospitals with NICU (level III) also had lower LTFU. These trends all lost significance if there was an audiologist involved in the programme.  Conversely, Scheepers et al. reported higher LTFU from the hospital with a higher birth rate [27]. The increased stress on screeners to screen all infants before discharge likely contributed to the higher referral rate and the higher LTFU rate reported from this hospital. |
| **Programme organization and tracking** | In South Korea, two pilot projects were carried out, an area-based programme and coupon-based programme [44]. In the area-based programme, designated clinics carried out screening independently, and aggregate results were submitted to the public health database. In the coupon-based programme, information was provided to parents before birith in addition to a coupon, which was submitted to designated clinics that carried out NHS. Individual-level data was managed by a central public health unit and tracking system. With a coupon system, referral rate was reduced from 2.5% to 1.4%  In Australia, Barker et al. found that 21.7% of NICU infants failed screening in the stand-alone programme compared to 3.9% when NICU screening was embedded into the universal NHS programme [45]. | | In the South Korea study, a coupon-based system with centralized tracking reduced LTFU from 87.6% to 66.5% [44]. Barker et al. showed an improvement in LTFU when NICU screening was embedded into a universal NHS programme [45]. |
| **Step 2 booking procedure** |  |  | The requirement for parents to pay a step 2 screening fee was associated with higher LTFU [38]. Thomson and Yoshinaga-Itano found a significantly higher LTFU rate when parents were responsible for booking the step 2 screening, compared to if the booking was done prior to hospital discharge; however, this variable was directly related to whether an audiologist was involved in the NHS programme. When audiologist involvement was accounted for in the model, the effect of the step 2 screening appointment was reduced [3]. |
| **Compliance with guidelines** |  |  | No association was found [38]. |
| **Referral rate step 1** |  |  | It is difficult to determine whether lower referral rate from screening step 1 will also result in a lower LTFU rate due to the possible interactions between the programme variable compared. Thomson and Yoshinaga-Itano showed that the involvement of an audiologist in the screening programme removed any associative trend between referral and LTFU rates [3]. |
| **Step 2 location** |  |  | Hospitals that referred families to an external audiology clinic for step 2 screening had a higher LTFU rate compared to if families returned to the initial screening location. LTFU was even lower if the screening hospital had an audiology department [3]. No association was found by Cunningham et al. [38]. In Barker et al., no NICU infants were LTFU when returning for step 2 at the screening hospital, compared to 14.3% of infants that were scheduled directly for diagnostic assessment. Out of the infants referred from step 2 to diagnostic assessment, 4% were LTFU to diagnostic assessment [45].  In a controlled intervention study among lower-income families [46], LTFU among WB was significantly lower when step 2 screening was implemented at accessible Women, Infants and Children’s locations, compared to control groups where infants that failed step 1 would return to the hospital for follow-up. |

1. Finitzo, T., K. Albright, and J. O'Neal, *The newborn with hearing loss: detection in the nursery.* Pediatrics, 1998. **102**(6): p. 1452-1460.

2. Lin, H.C., et al., *reducing false positives in newborn hearing screening program: how and why.* Otology & Neurotology, 2007. **28**(6): p. 788-792.

3. Thomson, V. and C. Yoshinaga-Itano, *The Role of Audiologists in Assuring Follow-Up to Outpatient Screening in Early Hearing Detection and Intervention Systems.* American Journal of Audiology, 2018. **27**(3): p. 283-293.

4. Govaerts, P.J., et al., *A Two-stage bipodal screening model for universal neonatal hearing screening.* Otology & neurotology : official publication of the American Otological Society, American Neurotology Society [and] European Academy of Otology and Neurotology, 2001. **22**(6): p. 850-854.

5. Murray, G., et al., *Evaluation of the Natus ALGO 3 Newborn Hearing Screener.* JOGNN - Journal of Obstetric, Gynecologic, & Neonatal Nursing, 2004. **33**(2): p. 183-190.

6. Chan, K.T., et al., *Improving newborn hearing screening: Are automated auditory brainstem response ear inserts an effective option?* International journal of pediatric otorhinolaryngology, 2015. **79**(11): p. 1920-1925.

7. Kishino, A., et al., *Comparison between MB11 BERAphone R and ALGO2e color for hearing screening in Japanese healthy newborns.* International Journal of Pediatric Otorhinolaryngology, 2021. **144**: p. 110673.

8. Deniz, H., A. Yazici, and M.E. Coskun, *Neonates Hearing Screening Results: A Comparison of Chirp and Click Stimuli with an Automated Auditory Brainstem Response Device.* Cyprus Journal of Medical Sciences, 2020. **5**(4): p. 313-316.

9. Gabbard, S.A., J.L. Northern, and C. Yoshinaga-Itano, *Hearing screening in newborns under 24 hours of age.* Seminars in Hearing, 1999. **20**(4): p. 291-305.

10. Korres, S., et al., *The effect of different 'pass-fail' criteria on the results of a newborn hearing screening program.* Orl; Journal of Oto-Rhino-Laryngology & its Related Specialties, 2003. **65**(5): p. 250-253.

11. Korres, S., et al., *Overcoming difficulties in implementing a universal newborn hearing screening program.* Turkish Journal of Pediatrics, 2005. **47**(3): p. 203-212.

12. De Ceulaer, G., et al., *Neonatal hearing screening with transient evoked otoacoustic emissions: a learning curve.* Audiology : official organ of the International Society of Audiology, 1999. **38**(6): p. 296-302.

13. Vohr, B.R., et al., *Factors affecting the interpretation of transient evoked otoacoustic emission results in neonatal hearing screening.* Seminars in Hearing, 1993. **14**(1): p. 57-72.

14. Norton, S.J., et al., *Identification of neonatal hearing impairment: evaluation of transient evoked otoacoustic emission, distortion product otoacoustic emission, and auditory brain stem response test performance.* Ear & Hearing, 2000. **21**(5): p. 508-528.

15. Sininger, Y.S., et al., *Identification of neonatal hearing impairment: auditory brain stem responses in the perinatal period.* Ear & Hearing, 2000. **21**(5): p. 383-399.

16. Burdzgla, I., et al., *The proper time for hearing screening in newborns.* Georgian Medical News, 2007(144): p. 24-27.

17. Pastorino, G., et al., *The Milan Project: a newborn hearing screening programme.* Acta paediatrica (Oslo, Norway : 1992), 2005. **94**(4): p. 458-463.

18. Clemens, C.J. and S.A. Davis, *Minimizing false-positives in universal newborn hearing screening: a simple solution.* Pediatrics, 2001. **107**(3): p. E29.

19. Shoup, A.G., et al., *The Parkland Memorial Hospital experience in ensuring compliance with Universal Newborn Hearing Screening follow-up.* Journal of Pediatrics, 2005. **146**(1): p. 66-72.

20. Arslan, S., et al., *Universal newborn hearing screening; automated transient evoked otoacoustic emissions.* B-Ent, 2013. **9**(2): p. 122-131.

21. Berninger, E. and B. Westling, *Outcome of a universal newborn hearing-screening programme based on multiple transient-evoked otoacoustic emissions and clinical brainstem response audiometry.* Acta Oto-Laryngologica, 2011. **131**(7): p. 728-739.

22. Dimitriou, A., et al., *The universal newborn hearing screening program in a public hospital: The importance of the day of examination.* International Journal of Pediatric Otorhinolaryngology, 2016. **91**: p. 90-93.

23. Hrncic, N., et al., *Does an early discharge of a newborn influence the success of the newborn hearing screening in developing countries? A hospital based study.* Medicinski Glasnik Ljekarske Komore Zenickodobojskog Kantona, 2019. **16**(2): p. 01.

24. Korres, S., et al., *Otoacoustic emissions in universal hearing screening: which day after birth should we examine the newborns?* Orl; Journal of Oto-Rhino-Laryngology & its Related Specialties, 2003. **65**(4): p. 199-201.

25. Tabrizi, A.G., et al., *BIRTH BY CESAREAN DELIVERY ON NEWBORN HEARING SCREENING TEST: A RETROSPECTIVE STUDY.* International Journal of Life Science and Pharma Research, 2017. **7**(4): p. L26-L29.

26. Wessex Universal Neonatal Hearing Screening Trial Group, *Controlled trial of universal neonatal screening for early identification of permanent childhood hearing impairment.* The Lancet, 1998. **352**(9145): p. 1957-1964.

27. Scheepers, L.J., W. Swanepoel de, and T. Roux, *Why parents refuse newborn hearing screening and default on follow-up rescreening--a South African perspective.* International Journal of Pediatric Otorhinolaryngology, 2014. **78**(4): p. 652-658.

28. Vernier, L.S., et al., *Delivery Route and the Outcome of Newborn Hearing Screening of Full-Term Neonates Born in a Public Maternal-Infant Hospital in the South of Brazil.* International Archives of Otorhinolaryngology, 2021.

29. Chung, Y.S., S.H. Oh, and S.K. Park, *Referral rates for newborn hearing screening based on the test time.* International Journal of Pediatric Otorhinolaryngology, 2019. **127**: p. 109664.

30. Kelly, A.F., P.K. Kelly, and M. Shah, *Auditory Brainstem Response Pass Rates Correlate with Newborn Hour of Life and Delivery Mode.* Journal of Pediatrics, 2021. **230**: p. 100-105.

31. Labaeka, A.A., et al., *Prevalence of Hearing Impairment Among High-Risk Newborns in Ibadan, Nigeria.* Frontiers in Pediatrics, 2018. **6**: p. 194.

32. Olusanya, B.O., et al., *Costs and performance of early hearing detection programmes in Lagos, Nigeria.* Transactions of the Royal Society of Tropical Medicine & Hygiene, 2009. **103**(2): p. 179-186.

33. Kanji, A., et al., *Feasibility of infant hearing screening from a developing country context: the South African experience.* Hearing, Balance & Communication, 2018. **16**(4): p. 263-270.

34. Kolski, C., et al., *Early hearing screening: what is the best strategy?* International Journal of Pediatric Otorhinolaryngology, 2007. **71**(7): p. 1055-1060.

35. Uilenburg, N., et al., *An implementation study of neonatal hearing screening in the Netherlands.* International Journal of Audiology, 2009. **48**(3): p. 108-116.

36. de Kock, T., D. Swanepoel, and J.W. Hall, 3rd, *Newborn hearing screening at a community-based obstetric unit: Screening and diagnostic outcomes.* International Journal of Pediatric Otorhinolaryngology, 2016. **84**: p. 124-131.

37. Stewart, D.L., et al., *Universal newborn hearing screening with automated auditory brainstem response: a multisite investigation.* Journal of perinatology : official journal of the California Perinatal Association, 2000. **20**(8 Pt 2): p. S128-31.

38. Cunningham, M., et al., *Infant, Maternal, and Hospital Factors' Role in Loss to Follow-up After Failed Newborn Hearing Screening.* Academic pediatrics, 2018. **18**(2): p. 188-195.

39. Olusanya, B.O., *Ambient noise levels and infant hearing screening programs in developing countries: an observational report.* International Journal of Audiology, 2010. **49**(8): p. 535-541.

40. Hergils, L., *Analysis of measurements from the first Swedish universal neonatal hearing screening program.* International Journal of Audiology, 2007. **46**(11): p. 680-685.

41. Mehl, A.L. and V. Thomson, *The Colorado newborn hearing screening project, 1992-1999: on the threshold of effective population-based universal newborn hearing screening.* Pediatrics, 2002. **109**(1): p. E7.

42. Fan, J.Y., et al., *A pre-paid newborn hearing screening programme: a community-based study.* B-ENT, 2010. **6**(4): p. 265-269.

43. Prince, C.B., et al., *Epidemiology of early hearing loss detection in Hawaii.* Pediatrics, 2003. **111**(5 Pt 2): p. 1202-1206.

44. Park, S.K., et al., *Analysis of the effectiveness of coupon-mediated newborn hearing screening program through comparison of two government-funded pilot projects in South Korea.* International Journal of Pediatric Otorhinolaryngology, 2020. **136**: p. 110256.

45. Barker, M.J., E.K. Hughes, and M. Wake, *NICU-only versus universal screening for newborn hearing loss: Population audit.* Journal of Paediatrics & Child Health, 2013. **49**(1): p. E74-79.

46. Hunter, L.L., et al., *Influence of the WIC Program on Loss to Follow-up for Newborn Hearing Screening.* Pediatrics, 2016. **138**(1): p. 07.
